# Supplementary material for: Mortality during 6 years of follow-up in relation to visual impairment and eye disease: results from a population-based cohort study of people aged 50 years and above in Nakuru, Kenya
Source: BMJ Open. 2019 Jun 9;9(6):e029700. doi: 10.1136/bmjopen-2019-029700 (PMC6561440; doi:10.1136/bmjopen-2019-029700)
Supplement: Supplementary Table 1 [file bmjopen-2019-029700supp001.pdf]

Web Table 1. 6-Year adjusted mortality risk by level of VI among the Nakuru Eye Disease Cohort Study Participants, stratified by age and gender (Unweighted)

	Overall		Male		Female		<60 years		≥60 years	
	N	Risk per 1,000/6yrs (95%CI)	N	Risk per 1,000/6yrs (95%CI)	N	Risk per 1,000/6yrs (95%CI)	N	Risk per 1,000/6yrs (95%CI)	N	Risk per 1,000/6yrs (95%CI)
Visual acuity at baseline (better eye presenting)										
All individuals	409 / 3441	119(106,134)	236 / 1656	143(124,164)	173 / 1785	97(83,113)	86 / 1503	57(46,71)	323 / 1938	167(150,185)
Normal (≥6/12)	280 / 2901	97(84,111)	162 / 1378	118(98,140)	118 / 1523	78(64,93)	78 / 1420	55(44,69)	202 / 1481	136(118,157)
Near Normal (<6/12-≥6/18)	27 / 170	159(109,226)	14 / 84	167(104,256)	13 / 86	151(87,250)	3 / 27	111(34,309)	24 / 143	168(115,238)
VI (<6/18-≥6/60)	77 / 275	280(234,331)	45 / 142	317(252,390)	32 / 133	241(172,325)	3 / 31	97(30,268)	74 / 244	303(250,363)
SVI (<6/60-≥3/60)	4 / 16	250(83,552)	2 / 10	200(37,622)	2 / 6	333(42,851)	2 / 2	-	2 / 14	143(28,490)
Blind (<3/60)	19 / 50	380(242,541)	13 / 30	433(246,642)	6 / 20	300(129,553)	0 / 6	-	19 / 44	433(275,604)
Any VI (<6/18)	100 / 341	293(245,347)	60 / 182	330(268,398)	40 / 159	252(184,333)	5 / 39	128(52,281)	95 / 302	315(260,375)
