# Supplementary material for: Mortality during 6 years of follow-up in relation to visual impairment and eye disease: results from a population-based cohort study of people aged 50 years and above in Nakuru, Kenya
Source: BMJ Open. 2019 Jun 9;9(6):e029700. doi: 10.1136/bmjopen-2019-029700 (PMC6561440; doi:10.1136/bmjopen-2019-029700)
Supplement: Author's manuscript [file bmjopen-2019-029700.draft_revisions.pdf]

BMJ Open is committed to open peer review. As part of this commitment we make the peer review history of every article we publish publicly available.

When an article is published we post the peer reviewers' comments and the authors' responses online. We also post the versions of the paper that were used during peer review. These are the versions that the peer review comments apply to.

The versions of the paper that follow are the versions that were submitted during the peer review process. They are not the versions of record or the final published versions. They should not be cited or distributed as the published version of this manuscript.

BMJ Open is an open access journal and the full, final, typeset and author-corrected version of record of the manuscript is available on our site with no access controls, subscription charges or pay-per-view fees (http://bmjopen.bmj.com).

If you have any questions on BMJ Open's open peer review process please email info.bmjopen@bmj.com

BMJ Open

Six-year mortality risk in relation to visual impairment and eye disease: Results from a population-based cohort study of people aged 50 years and above in Nakuru, Kenya

Journal:	*BMJ Open*
Manuscript ID	bmjopen-2019-029700
Article Type:	Research
Date Submitted by the Author:	07-Feb-2019

Complete List of Authors:	Kuper, Hannah; The London School of Hygiene & Tropical Medicine, Clinical Research<br>Mathenge, Wanjiku; Univ London London Sch Hyg<br>Macleod, David; Univ London London Sch Hyg<br>Foster, Allen; Univ London London Sch Hyg<br>Gichangi, Michael; Division of Preventive Ophthalmic Services, Ministry of Public Health and Sanitation<br>Rono, Hillary; Univ London London Sch Hyg<br>Wing, Kevin; London School of Hygiene and Tropical Medicine,<br>Weiss, Helen; London School of Hygiene and Tropical Medicine,<br>Burton, Matthew J; London School of Hygiene and Tropical Medicine<br>Bastawrous, Andrew; Univ London London Sch Hyg

Keywords:	blindness, Kenya, mortality

SCHOLARONE™
Manuscripts

Six-year mortality risk in relation to visual impairment and eye disease: Results from a population-based cohort study of people aged 50 years and above in Nakuru, Kenya

Authors: Hannah Kuper, ScD[1,2]*, Wanjiku Mathenge, PhD[3], David Macleod, MSc[4], Allen Foster, FRCS[1,2], Michael Gichangi, MSc[5], Hillary Rono, MSc[1,6], Kevin Wing, PhD[7], Helen A Weiss, DPhil[4], Matthew Burton, PhD[1], Andrew Bastawrous, PhD[1]

*Corresponding author: Professor Hannah Kuper, London School of Hygiene and Tropical Medicine, London, United Kingdom. E-mail: Hannah.kuper@lshtm.ac.uk

[1]International Centre for Eye Health, Clinical Research Department, London School of Hygiene and Tropical Medicine, London, United Kingdom

[2]International Centre for Evidence in Disability, Clinical Research Department, London School of Hygiene and Tropical Medicine, London, United Kingdom

[3]Rwanda International Institute of Ophthalmology and Dr. Agarwal's Eye Hospital, Kigali, Rwanda

[4]MRC Tropical Epidemiology Group, Department of Infectious Disease Epidemiology, London School of Hygiene & Tropical Medicine, London, United Kingdom

[5]Ministry of Health, Nairobi, Kenya

[6]Kitale Eye Unit, Ministry of Health Trans Nzoia County, Kenya

[7] Department of Non-Communicable Disease Epidemiology, London School of Hygiene & Tropical Medicine, London, United Kingdom

Manuscript word count: 3139

For peer review only

Abstract

Objective: To estimate the association between (1) visual impairment (VI) and (2) eye disease and 6-year mortality risk within a cohort of elderly Kenyan people.

Design, setting and participants: The baseline of the Nakuru Posterior Segment Eye Disease Study was formed from a population-based survey of 4318 participants aged ≥50 years, enrolled in 2007-2008. Detailed ophthalmic and anthropometric examinations were undertaken on all participants at baseline, and a questionnaire was administered, including past medical and ophthalmic history. Participants were retraced in 2013-2014 for a second examination phase. Vital status was recorded for all participants through information from community members. Cumulative incidence of mortality, and its relationship with baseline VI and types of eye disease was estimated. Inverse probability weighting was used to adjust for nonparticipation.

Primary outcome measures: Cumulative incidence of mortality in relation to visual impairment level at baseline.

Results: Of the baseline sample, 2,170 (50%) were re-examined at follow-up and 407 (10%) were known to have died. Compared to those with normal vision (visual acuity (VA) ≥6/12), the 6-year mortality risk was higher among people with VI (<6/18-≥6/60; RR=1.75, 1.28-2.40) or severe VI/blindness (<6/60; RR=1.98, 1.04-3.80). These associations remained after adjustment for Non Communicable Disease (NCD) risk factors (mortality: RR=1.56, 95% CI 1.14-2.15; SVI/blind: RR=1.46, 95% CI 0.80-2.68). Mortality risk was also associated with presence of diabetic retinopathy at baseline (RR=3.18, 95% CI=1.98-5.09), cataract (RR=1.26, 0.95-1.66), and presence of both cataract and VI (RR=1.57, 1.24-1.98). Mortality risk was higher among people with age-related macular degeneration at baseline (with or without VI), compared with those without (RR=1.42, 0.91-2.22 and RR=1.34, 0.99-1.81, respectively).

Conclusions: Visual acuity was related to six-year mortality risk in this cohort of elderly Kenyan people, potentially because both VI and mortality are related to ageing and risk factors for NCD.

Strengths and limitations of this study

- The cohort comprised of a representative population-based sample in an area of ethnic, socioeconomic, and educational diversity.
- There was comprehensive assessment of ophthalmic characteristics and risk factors at baseline and follow-up.
- Data on mortality was collected through informant report, rather than from death certificates.
- There was a high loss to follow-up in this study, raising the possibility of selection bias.

Funding/Support: This study was supported by grant G1001934 from the Medical Research Council, grant 1310 from Fight for Sight, the British Council for the Prevention of Blindness, and the International Glaucoma Association (Dr Bastawrous). Dr Burton is supported by grant 098481/Z/12/Z from the Wellcome Trust. Dr Weiss is supported by grant G0700837 from the Medical Research Council and Department for International Development. The funding sources had no role in the design and conduct of the study; collection, management, analysis, and interpretation of the data; preparation, review, or approval of the manuscript; and the decision to submit the manuscript for publication.

Author Contributions: Professor Kuper had full access to all the data in the study and takes responsibility for the integrity of the data and the accuracy of the data analysis.

- *Study concept and design:* Bastawrous, Mathenge, Foster, Burton, Kuper.
- *Acquisition, analysis, or interpretation of data:* Bastawrous, Wing, Rono, Weiss, Macleod, Burton.
- *Drafting of the manuscript:* Kuper, Bastawrous, Macleod.
- *Critical revision of the manuscript for important intellectual content:* Bastawrous, Mathenge, Gichangi, Wing, Rono, Weiss, Foster, Burton, Kuper.
- *Statistical analysis:* Kuper, Bastawrous, Wing, Weiss, Macleod.
- *Obtained funding:* Bastawrous, Kuper.

For peer review only

- *Administrative, technical, or material support:* Bastawrous, Mathenge, Gichangi, Rono, Kuper.
- *Study supervision:* Bastawrous, Foster, Burton, Kuper.

Conflict of Interest: None.

Data sharing: Data is available on request from Andrew Bastawrous or Hannah Kuper.

For peer review only

Introduction

Visual impairment (VI) is common, affecting approximately 253 million people globally. [1] It can impact on different aspects of people's lives, including reducing quality of life, and increasing poverty and depression. [2-5] There is growing evidence from Europe, North America, Asia and Australia that VI and specific eye conditions are linked to increased risk of mortality,[6-17] but data are lacking for Low and Middle Income Countries (LMIC), particularly from Sub-Saharan Africa.

There are several potential pathways by which VI may be linked with mortality. Both VI and mortality are related to ageing, and so confounding or residual confounding may explain the reported associations. There are also common underlying risk factors for both VI and mortality, such as smoking, obesity, and poverty. For instance, VI due to age-related macular degeneration (AMD) is more common among smokers, [18] and smoking increases risk of mortality. An underlying disease may also cause both VI and mortality, for instance diabetic retinopathy (DR) is related to poor control of diabetes, which also causes increased mortality. People with VI may find it more difficult to seek health-care, due to a range of barriers,[19] thereby increasing their mortality risk. Changes in the eye may be a marker of ageing, or accelerated ageing,[20] and thereby linked to mortality. Finally, VI could acerbate frailty, depression and functional difficulties, all linked to increased mortality. [3 21 22]

It is important to explore whether there is an association between VI and mortality and, if there is, to identify possible pathways for this link, in order to understand how to reduce the vulnerability of people with VI to increased morbidity and mortality. Furthermore, these data may be useful to advocate for scaling up of eye health services in LMICs. The objective of this study was to investigate the association between VI and six-year mortality risk within the Nakuru Eye Disease Cohort Study, a cohort of elderly Kenyan people.

For peer review only

Materials and Methods

The methodology of the Nakuru Eye Disease Cohort Study has been reported previously,[23] and is summarised here.

Baseline Study Population

The baseline population based survey was conducted in 2007/8. A total of 100 clusters each of 50 participants were selected with a probability proportional to the size of the population across Nakuru district. Households were selected within clusters using a modified compact segment sampling method.[24] Eligible individuals were those aged ≥50 years living in the household for at least three months in the previous year, and multiple subjects could be included per household.

Baseline Ophthalmic and General Examination

All participants were invited to undergo a comprehensive ophthalmic examination at a screening clinic. [23] The objectives of the survey and the examination process were explained to those eligible in the local dialect, in the presence of a witness. A subject was examined only after informed written (or thumbprint) consent was obtained.

All participants underwent logMAR visual acuity testing on each eye separately and corrected visual acuity (by refraction or pinhole) when less than 20/40 Snellen equivalent. Participants had 2 non-stereoscopic, digital, 45° fundus photographs (1 disc and 1 macula centered) taken per eye by an ophthalmic clinical officer. Digital images were graded for the presence of AMD and DR at an approved grading center (Moorfields Eye Hospital Reading Centre) by a senior grader, with adjudication by a clinician for confirmed cases and 5% of randomly selected images to ensure quality control. The presence of cataract was recorded by the ophthalmologist (WM) on slit-lamp examination after pupil dilation.

Detailed interviews were undertaken in the local language on demographic details, information on risk factors, socioeconomic status (SES), and full medical history.

A nurse performed and recorded measures of participants: height (Leicester Height Measure); weight (Seca 761); and three measures of blood pressure (Omron® Digital Automatic Blood Pressure Monitor Model HEM907), each ten minutes apart. Capillary blood was taken from all participants for random blood glucose (Accutrend GC system).

For peer review only - http://bmjopen.bmj.com/site/about/guidelines.xhtml

Assessment of vital status at follow-up

Follow-up was conducted from January 2013 to March 2014. A meeting was held approximately one week before the follow-up examination clinic for a given cluster. A list of study participants was given to the chief and a local village guide was recruited to assist location of the study participants. The village guide was someone who knew and was well known by the community (or the village chief him/herself). The Advance Team visited homes of baseline participants on the day prior to the examination clinic and confirmed their identity using National Identity cards and invited them to attend the examination clinic the following day. All identified participants were also asked to help locate baseline participants that had not been found.

Each local field guide was asked to classify the baseline study participant for that cluster as "available", "died", "moved away" or "unknown". A participant was defined to have died if this was verified by at least two people from amongst the village chief, local guide or available study participant. Those who were known to have moved away were contacted when possible to either arrange follow-up at a more suitable location for the participant or to identify if they were alive or had died in the follow-up period. Any participant for whom nobody could identify as being alive or having moved away was recorded as "unknown".

Definitions and Statistical Analyses

All participants who had complete examinations at baseline were considered "at-risk" for mortality during follow up. Follow-up status at 6 years was categorised as:

i) Deceased (confirmed dead, as described above);
ii) Alive (i.e. re-examined at follow-up, retraced but refused or unavailable at follow-up, or moved away but known to be alive)
iii) Unknown (i.e. not retraced at follow-up, death not verified as described above, or moved away but vital status unknown).

A socioeconomic status (SES) score was developed based on information collected on job, housing conditions, and ownership of material goods and livestock, based on previous work in the same population. [25] Hypertension was defined on the basis of the average of the second and third reading, with cut-offs used of systolic blood pressure≥140 mmHG and/or diastolic blood

For peer review only - http://bmjopen.bmj.com/site/about/guidelines.xhtml

pressure≥90 mmHG and/or self-reported hypertension medication. Diabetes was defined as (1) Self-reported in the history, or (2) random glucose of ≥11.0mmol/L.

Statistical analysis was performed using STATA v14 (Stata Corp). All analyses accounted for the cluster survey design using Taylor linearized variance estimation to calculate standard errors. Pearson Chi-squared tests corrected for the survey-design were used to calculate p-values to assess differences between participants whose mortality status is known and those where mortality status is unknown, i.e. lost to follow-up (LTFU).

An inverse probability-weighting (IPW) model[26] was developed, in order to allow estimation of mortality risk while accounting for those LTFU. Multivariable logistic regression was used to identify independent baseline covariates associated with LTFU. Covariates for which there was evidence of univariable association with the outcome ($p<0.10$ across all categories of the variable) were kept in a multivariable model (age, sex, rural/urban and mother tongue). From this final model, the probability of being followed up was estimated, based on the presence or absence of each of these baseline covariates. The inverse of this probability formed the weighting to be applied to account for those LTFU.

The final step was to remove those individuals LTFU from the cohort, so that all subsequent analysis would be performed on only those with complete outcome records, with IPW applied to account for those LTFU. A sensitivity analyses for this approach involved repeating the analyses without applying IPW (i.e. standard unweighted complete case analysis), and assessing the impact on the results.

Six-year mortality risk was calculated by dividing the number of deaths identified at follow-up by the number of people at risk at baseline. 95% confidence intervals were estimated assuming a Poisson distribution of events. This was done for the population overall, and stratified by each covariate.

Age/sex-adjusted risk ratios for each covariate in relation to mortality were estimated using a Poisson regression model with robust error variance to allow for the clustered design and including IPW. Mortality status was the binary outcome and the distribution was assumed to be Poisson. These analyses were adjusted for the clustered design, as well as the use of IPW, by setting the clusters as the primary sampling unit and weighting using the inverse probability of being followed-up. The model was further adjusted using a set of four socioeconomic (SES) variables only (SES quartile, location, ethnic group, education), then a set of five non-

For peer review only - http://bmjopen.bmj.com/site/about/guidelines.xhtml

communicable disease (NCD) risk factors only (smoking, alcohol, diabetes, hypertension, BMI) before finally adjusting for all nine SES and NCD variables.

Patient and Public Involvement

Patients and the public were not directly involvement in the development of the research question and outcome measures, or the design or conduct of study. There are no plans to disseminate results directly to the study participants.

Ethical Approval

The study adhered to the tenets of the Declaration of Helsinki and was approved by the Ethics Committee of London School of Hygiene and Tropical Medicine at both baseline and follow-up (LSHTM Ref 6192). Baseline approval was provided by the Kenya Medical Research Institute Ethics Committee and by the African Medical and Research Foundation (AMREF) Ethics Committee, Kenya for the follow-up (AMREF-ESRC P44/12). For both phases, approval was granted by the Rift Valley Provincial Medical Officer and the Nakuru District Medical Officer of Health. Approval was sought from the administrative heads in each cluster.

For peer review only

Results

At baseline 4,414 participants were examined. The follow-up assessment was conducted, on average, 5.6 years (SD 0.6) after the baseline, expressed for simplicity as 6 years (meaning that there was 6 years between the baseline and follow-up wave, rather than that each participant was followed-up on average for 6 years as the time of loss to follow-up or death was not known for individuals). Of the baseline participants, 3032 were known to be alive at follow-up (69% 2,170 re-examined at the follow-up plus 862 known to be alive but not re-examined), 409 (9%) were known to have died, and 973 had unknown vital status (22%)..

Table 1 provides the baseline characteristics of participants who had died during the follow-up, those were re-examined at follow-up and those who were LTFU. In comparison to those who had died, those who were re-examined were younger, more likely to be female, Kalenjin speakers, and had higher SES, while those of unknown status were more likely to be of "other" tribes and urban residence.

Table 2 shows the weighted 6-year mortality risk by level of VI. Overall, the 6-year mortality risk increased with worsening levels of VI, from 97/1000 (95% CI 84-111/1000) among those with normal vision to 385/1000 (245-548/1000) among those who were blind. This pattern was observed in both males and females, but was less clear in people aged <60 years given the low mortality in this group and consequent small numbers. In each sub-group, the lowest risk of mortality was among people with normal vision. Mortality risk among people with VI was higher for males than for females, and among those ≥60 years versus <60 years. Estimates changed little after weighting for LTFU (web table for unweighted estimates).

Compared to those with normal vision (VA>6/12), the mortality risk was significantly higher among people with VI (VA<6/18-≥6/60; RR=1.75, 95% CI 1.28-2.40) or SVI/blindness (VA<6/60: RR=1.98, 1.04-3.80) (Table 3). There was a weakening of the association after adjustment for non-communicable disease (NCD) risk factors or full adjustment for both socio-economic status (SES) and NCD risk factors, although the overall trends between worsening vision and increased 6-year mortality risk remained evident (VI: RR=1.56, 95% CI 1.14-2.15; SVI/blind: RR=1.46, 95%CI 0.80-2.68).

People with any VI had a higher mortality risk than those without VI (RR=1.54, 95%CI 1.22-1.93), and this association remained after adjustment for SES and NCD risk factors (RR=1.37, 95%CI 1.10-1.71) (Table 4). Other risk factors associated with 6-year mortality risk after comprehensive adjustment included increasing age (oldest versus youngest age group: RR=4.68, 95%CI 3.55-6.18) and diabetes (RR=2.34, 95%CI 1.81-3.03). Being underweight was associated with an increased 6-year mortality risk (underweight versus

For peer review only

normal: RR=1.60, 95%CI 1.24-2.07).

Risk of mortality was analysed by prevalence of specific eye diseases at baseline (Table 5). The presence of cataract (or aphakia/pseudophakia) and any VI (i.e. VA <6/18 in better eye) was associated with higher mortality risk (RR=1.57, 95%CI 1.24-1.98), whereas cataract alone (or aphakia/pseudophakia) was not (RR=1.26, 95%CI 0.95-1.66). Mortality risk was higher among people with AMD at baseline (with or without VI), compared with those without, although these associations were not statistically significant (RR=1.42, 95%CI 0.91-2.22 and RR=1.34, 95%CI 0.99-1.81, respectively). DR was associated with a three-fold increased mortality risk (RR=3.18, 95%CI 1.98-5.09). The number of people with DR and any VI were too small to make meaningful inferences.

For peer review only

Discussion

VI was associated with increased mortality risk during six years of follow-up in a cohort of elderly Kenyan people. The risk of mortality increased with worsening vision. This association was reduced after adjustment for the presence of NCD risk factors, and to a lesser extent for SES indicators. Among eye conditions, DR was most strongly associated with mortality risk, although the number affected was small. Cataract with VI was also associated with elevated mortality, as were AMD and cataract without visual loss at baseline (although these estimates lacked precision).

Previous studies have also shown a positive relationship between VI and mortality, with evidence available from the USA [6-9], UK[10], Australia[11 12], Japan [13], Singapore [14 15], China[16] and India [17]. Others have failed to find evidence for this association, including in India [27], Iceland [28], and Taiwan. [29] Data from LMICs are sparse, in particular for Sub-Saharan Africa, and so comparison of our study findings to those from similar settings is not possible.

On the basis of our findings and those in the wider literature consideration can be given to the potential pathway for the association between VI and mortality. There was clear evidence for confounding by age, as both VI and mortality are independently related to older age. Consequently, imperfect adjustment for age may have allowed for residual confounding as a partial explanation for the association. There was little evidence for confounding by SES, although in this setting high SES was associated with greater prevalence of NCD risk factors,[30] and a somewhat reduced mortality risk. The presence of NCD risk factors may also act as confounders of the association of VI on mortality, since the association was attenuated after adjustment for these indicators, as found in other studies.[10] Significant associations persisted, however, between VI and mortality after comprehensive multivariable adjustment in this study, as occurred in previous studies, [12-14 17 31] suggesting that residual confounding or direct effects of VI on mortality may be operational.

Exploring the relationship between different eye conditions and mortality may help to clarify whether independent biological pathways exist. DR is known to be associated with increased mortality,[15 32] as was also shown in this study. This link is unsurprising given the well-known relationship between uncontrolled diabetes with both DR and mortality. However, the relatively small number of people with DR in this population means that this link cannot be the

For peer review only - http://bmjopen.bmj.com/site/about/guidelines.xhtml

sole driver of the VI-mortality association. Our study as well as others have shown cataract to be associated with increased mortality. [6 12 17 33 34], though this association is not always demonstrated.[15 35] Some studies have suggested that this relationship varies by cataract type. [8 34 36-38] It is hypothesized that the association between cataract and mortality arises as lens opacification (cataract) is an indicator of accelerated ageing. [20 39] The evidence for a link between AMD and mortality is more complex; some studies show that late AMD is associated with mortality, but not early AMD. [6 40-42] Others found no association between AMD and mortality, [15 33 35] or only among women.[43]

There are other potential pathways between VI and mortality not explored in this study. For example, NCD risk factors may be mediators of the effect of VI on mortality (rather than confounders) for reasons such as lower ability to access NCD treatment, less exercise, poorer diets and so on. Consequently, the association between VI and mortality adjusted by NCD risk factors would be an underestimate of the total effect. There were also concerns about the accuracy of assessment of visual fields in this population. Consequently it was not possible to determine the presence of glaucoma at baseline, although others have suggested a link between glaucoma and mortality. [15 44] We also did not assess the impact of VI in accessing health care, although the Australian Blue mountain study showed that difficulties in walking explained some of the link between VI and mortality.[11] Only 18 people with cataract underwent cataract surgery during the follow-up period, so it was not possible to assess the impact on mortality.

There are several further limitations of the study, which need to be considered when interpreting the findings. There was a lack of data on date of death, and no verification from death certificates, as these are rarely available in many African settings, [45] including Kenya. Cause of death could not be determined, and so we could not assess whether the relationship was stronger between VI and specific causes of mortality, notably cardiovascular and non-cancer causes, as demonstrated in previous studies,[15 46-48] which would lend weight to a biological pathway for the association. The follow-up study was conducted after a period of post-election violence in the area. Consequently, there was a high loss to follow-up in this study, raising the possibility of selection bias influencing the findings, although patterns changed little after weighting for loss to follow up. Furthermore, the mortality rate may have been higher in this period, due to violence, and may have biased the association with VI if these deaths were disproportionally among people with VI, or among younger people (with lower prevalence of VI). Date of loss to follow-up or death was not recorded, and so survival analysis was not possible. Another concern is that reports of local informants was used to categorise some people who had moved away as “known to be alive”, which may have created

For peer review only - http://bmjopen.bmj.com/site/about/guidelines.xhtml

inaccuracies. The study may have been under-powered for some of the sub-group analyses, such as assessing the link between type of eye disease and mortality. We did not evaluate the association of different sub-types of AMD or cataract in relation to mortality because of small numbers.VI classification did not include loss of visual fields, and so the prevalence of functionally significant sight loss may have been underestimated. Self-reported diabetes was not confirmed (e.g. from medical records). In terms of strengths, this was the first study of its kind in sub-Saharan Africa to assess the association between VI and mortality. The study participants comprised a representative population-based sample in an area of ethnic, socioeconomic, and educational diversity. There was comprehensive assessment of ophthalmic characteristics and risk factors at baseline, and every attempt was made to follow up all participants, and to record vital status.

In conclusion, visual acuity was related to six-year mortality risk in this cohort of elderly Kenyan people. The most likely explanation for the association is that both VI and mortality are related to ageing and NCD risk factors. The implication is that continuity of care is needed, as people with VI require linkages to preventative and treatment services. Furthermore, we must advocate for the scale-up of eye care services in Kenya, as VI is linked to premature mortality.

For peer review only

Table 1. Baseline Characteristics of the Nakuru Eye Disease Cohort Study

**Baseline Characteristics**		*Deceased N=409*	*Followed-up or known to be alive n=3,032*	*p-value**	*Unknown status N=973*	*p-value ***
Age in years, mean (SD)		71.6 (12.8)	62.4 (9.6)	<0.001	63.2 (10.6)	0.60
Sex, % (n)	Female	42% (173)	53% (1,612)	<0.001	53% (516)	0.59
	Male	58% (236)	47% (1,420)		47% (457)	
Tribe, % (n)	Kikuyu	69%(283)	62% (1,881)	0.02	61% (596)	0.005
	Kalenjin	23% (93)	24% (736)		19% (186)	
	Other	8% (33)	14% (415)		20% (191)	
Education, % (n)***	None	6% (26)	9% (283)	<0.001	12% (114)	0.21
	Primary	44% (179)	32% (952)		33% (323)	
	Secondary	43% (174)	48% (1,448)		44% (427)	
	Higher	7% (29)	11% (327)		11% (105)	
Residence, % (n)	Rural	74% (303)	71% (2,167)	0.43	51% (498)	<0.001
	Urban	26% (106)	29% (865)		49% (475)	
SES Quartile, % (n)***	Lower	33% (136)	24% (709)	0.002	26% (247)	0.17
	Middle lower	24% (96)	26% (776)		23% (219)	
	Middle upper	24% (97)	26% (777)		23% (218)	
	Upper	19% (79)	24% (731)		29% (281)	

*p-value describes the strength of evidence that each variable is associated with mortality, among those where we know the mortality status (Null hypothesis is

For peer review only

For peer review only - http://bmjopen.bmj.com/site/about/guidelines.xhtml

that the odds of death are equal in each category of the variable)

**p-value describes the strength of evidence that each variable is associated with mortality status being missing (i.e. comparison of known versus unknown mortality status). (Null hypothesis is that odds of knowing the mortality status of an individual at follow up are equal in each group)

*** There were 27 missing values for education, and 48 missing values for SES.

For peer review only

Table 2. 6-Year weighted mortality risk by level of VI among the Nakuru Eye Disease Cohort Study Participants, stratified by age and gender

WEIGHTED USING IPWs	Overall		Male		Female		<60 years		≥60 years	
	N	Risk per 1,000/6yrs (95%CI)	N	Risk per 1,000/6yrs (95%CI)	N	Risk per 1,000/6yrs (95%CI)	N	Risk per 1,000/6yrs (95%CI)	N	Risk per 1,000/6yrs (95%CI)
Visual acuity at baseline (better eye presenting)										
All individuals	409 / 3441	119(106,134)	236 / 1656	143(123,165)	173 / 1785	98(84,113)	86 / 1503	56(45,70)	323 / 1938	169(151,188)
Normal (≥6/12)	280 / 2901	97(84,111)	162 / 1378	118(98,142)	118 / 1523	77(64,93)	78 / 1420	54(42,68)	202 / 1481	138(120,159)
Near Normal (<6/12-≥6/18)	27 / 170	158(107,226)	14 / 84	168(104,258)	13 / 86	148(85,245)	3 / 27	106(32,301)	24 / 143	168(114,241)
VI (<6/18-≥6/60)	77 / 275	283(236,336)	45 / 142	316(252,388)	32 / 133	249(178,338)	3 / 31	99(31,271)	74 / 244	307(252,368)
SVI (<6/60-≥3/60)	4 / 16	260(86,566)	2 / 10	187(34,603)	2 / 6	379(51,875)	2 / 2	-	2 / 14	136(27,475)
Blind (<3/60)	19 / 50	385(245,548)	13 / 30	436(247,646)	6 / 20	310(133,570)	0 / 6	-	19 / 44	438(279,612)
Any VI (<6/18)	100 / 341	297(248,351)	60 / 182	328(268,395)	40 / 159	262(191,347)	5 / 39	137(57,297)	95 / 302	318(262,380)

For peer review only

Table 3: Association between visual acuity category and 6-year mortality risk

VA category	Age-sex adjusted RR	Age-sex, SES* adjusted RR	Age-sex, NCD risk** factor adjusted RR	Fully adjusted*** RR
Normal (≥6/12)	Reference	Reference	Reference	Reference
Near Normal (<6/12-≥6/18)	0.92 (0.57 – 1.50)	0.84 (0.51 – 1.39)	0.87 (0.51 – 1.48)	0.82 (0.48 – 1.41)
VI (<6/18-≥6/60)	1.75 (1.28 – 2.40)	1.77 (1.30 – 2.40)	1.56 (1.13 – 2.16)	1.56 (1.14 – 2.15)
SVI/blind (<6/60)	1.98 (1.04 – 3.80)	1.95 (1.01 – 3.76)	1.51 (0.82 – 2.77)	1.46 (0.80 – 2.68)
p-value	0.004	0.003	0.04	0.03

*SES = SES quartile, location, ethnic group, education

**NCD risk factor = smoking, alcohol, diabetes, hypertension, BMI

***All SES and NCD risk factors

For peer review only

For peer review only - http://bmjopen.bmj.com/site/about/guidelines.xhtml

Table 4. Multivariable analysis of baseline co-variables and 6-year mortality risk in the Nakuru Eye Disease Cohort Study

	No at risk	Deaths	Risk per 1,000/6yrs (95%CI)	Age-sex-adjusted Risk Ratio	Age-sex-SES adjusted Risk Ratio	Age-Sex-SES-NCD risk factor adjusted Risk Ratio
Any VI (<6/18)						
- No	3071	307	100(87,115)	Reference	Reference	Reference
- Yes	341	100	297(248,351)	1.54(1.22,1.93)	1.55(1.24,1.94)	1.37(1.10,1.71)
Gender						
- Male	1656	236	143(123,165)	Reference	Reference	Reference
- Female	1785	173	98(84,113)	0.74(0.63,0.87)	0.68(0.56,0.83)	0.82(0.63,1.06)
Age						
- 50-59	1503	86	56(45,70)	Reference	Reference	Reference
- 60-69	1036	96	94(77,115)	1.64(1.24,2.17)	1.58(1.19,2.09)	1.40(1.07,1.83)
- 70-79	571	107	191(161,224)	3.27(2.55,4.20)	3.15(2.39,4.15)	2.74(2.09,3.60)
- 80+	331	120	363(311,419)	6.36(4.85,8.33)	5.76(4.39,7.57)	4.68(3.55,6.18)
**SES risk factors**						
Location						
- Rural	2470	303	123(108,140)	Reference	Reference	Reference
- Urban	971	106	110(83,145)	1.14(0.90,1.45)	1.15(0.90,1.48)	1.18(0.91,1.53)
SES quartile						
- Lower	845	136	164(137,197)	Reference	Reference	Reference

For peer review only

- Lower middle	872	96	110(91,134)	0.75(0.60,0.93)	0.72(0.58,0.91)	0.75(0.59,0.95)
- Upper middle	874	97	110(89,134)	0.86(0.66,1.11)	0.82(0.63,1.06)	0.84(0.65,1.09)
- Upper	810	79	99(78,126)	0.89(0.69,1.14)	0.78(0.58,1.05)	0.76(0.56,1.03)
Ethnic group						
- Kikuyu	2164	283	131(116,148)	Reference	Reference	Reference
- Kalenjin	829	93	113(91,139)	0.83(0.66,1.03)	0.78(0.62,0.99)	0.81(0.63,1.04)
- Other	448	33	76(48,119)	0.84(0.56,1.26)	0.82(0.55,1.21)	0.81(0.55,1.20)
Education						
- No education	309	26	86(58,125)	Reference	Reference	Reference
- Primary	1131	179	161(138,188)	0.95(0.65,1.41)	0.96(0.63,1.46)	0.97(0.63,1.50)
- Secondary	1622	174	107(91,125)	0.85(0.59,1.22)	0.84(0.57,1.24)	0.87(0.59,1.28)
- College/ Uni	356	29	81(52,124)	0.94(0.57,1.55)	0.95(0.57,1.55)	1.01(0.62,1.64)
**Risk factors for NCD**						
Smoking						
- Never	2396	256	107(92,124)	Reference	Reference	Reference
- Former	252	33	131(93,180)	1.20(0.85,1.69)	1.20(0.85,1.71)	1.15(0.79,1.66)
- Current	775	120	156(132,184)	1.19(0.93,1.52)	1.17(0.91,1.50)	1.19(0.91,1.55)
Diabetes						
- No	3202	354	111(98,125)	Reference	Reference	Reference
- Yes	216	54	248(194,313)	2.16(1.69,2.77)	2.20(1.69,2.84)	2.34(1.81,3.03)
Hypertension						

For peer review only - http://bmjopen.bmj.com/site/about/guidelines.xhtml

- No	1670	179	108(92,126)	Reference	Reference	Reference
- Yes	1737	229	132(113,153)	1.08(0.90,1.29)	1.06(0.88,1.28)	1.11(0.92,1.34)
Alcohol						
- Never	1335	117	87(73,104)	Reference	Reference	Reference
- Former	1520	221	147(125,171)	1.18(0.95,1.48)	1.18(0.94,1.49)	1.15(0.90,1.46)
- Current	559	70	125(100,157)	1.15(0.86,1.53)	1.18(0.87,1.60)	1.08(0.77,1.51)
BMI						
- Underweight	468	99	216(169,271)	1.55(1.20,2.00)	1.57(1.22,2.02)	1.60(1.24,2.07)
- Normal	1697	199	117(101,136)	Reference	Reference	Reference
- Overweight	779	66	86(66,111)	0.90(0.69,1.18)	0.87(0.66,1.14)	0.81(0.62, 1.05)
- Obese	447	32	73(52,100)	0.89(0.63,1.25)	0.86(0.59,1.24)	0.83(0.57,1.20)

For peer review only - http://bmjopen.bmj.com/site/about/guidelines.xhtml

Table 5: Risk of mortality during 6 years of follow up by the presence of specific eye diseases at baseline

	No at risk	Deaths	Risk per 1,000/6yrs (95%CI)	Age-sex-adjusted Risk Ratio
Cataract present				
- No	1921	142	72.5(59.1,88.6)	Reference
- Yes	1478	265	181.3(161.6,202.8)	1.26(0.95,1.66)
Cataract and VI present (<6/18)				
- No	3103	313	100.7(87.5,115.5)	Reference
- Yes	296	94	322(267.3,381.8)	1.57(1.24,1.98)
AMD present				
- No	2270	225	99.2(83.3,117.8)	Reference
- Yes	319	57	183.4(141.3,234.5)	1.34(0.99,1.81)
AMD and VI present (<6/18)				
- No	2529	265	105.5(89.9,123.4)	Reference
- Yes	60	17	282.7(178.5,416.8)	1.42(0.91,2.22)
DR present				

For peer review only

- No	2513	264	105.7(90.4,123.3)	Reference
- Yes	55	18	318.4(199.4,467.0)	3.18(1.98,5.09)
DR and VI present (<6/18)				
- No	2563	280	109.9(94.1,128.0)	Reference
- Yes	5	2	401.6(37.5,920.4)	2.54(0.57,11.36)

For peer review only

References

1. Bourne RRA, Flaxman SR, Braithwaite T, et al. Magnitude, temporal trends, and projections of the global prevalence of blindness and distance and near vision impairment: a systematic review and meta-analysis. *Lancet Glob Health* 2017;5(9):e888-e97. doi: 10.1016/S2214-109X(17)30293-0 [published Online First: 2017/08/07]
2. Banks LM, Polack S. The Economic Costs of Exclusion and Gains of Inclusion of People with Disabilities. London: International Centre for Evidence in Disability, 2014.
3. Ribeiro MV, Hasten-Reiter Junior HN, Ribeiro EA, et al. Association between visual impairment and depression in the elderly: a systematic review. *Arq Bras Oftalmol* 2015;78(3):197-201. doi: 10.5935/0004-2749.20150051 [published Online First: 2015/07/30]
4. Polack S, Kuper H, Mathenge W, et al. Cataract visual impairment and quality of life in a Kenyan population. *The British journal of ophthalmology* 2007;91(7):927-32. doi: 10.1136/bjo.2006.110973 [published Online First: 2007/02/03]
5. Polack S, Kuper H, Wadud Z, et al. Quality of life and visual impairment from cataract in Satkhira district, Bangladesh. *The British journal of ophthalmology* 2008;92(8):1026-30. doi: 10.1136/bjo.2007.134791 [published Online First: 2008/07/26]
6. Clemons TE, Kurinij N, Sperduto RD. Associations of mortality with ocular disorders and an intervention of high-dose antioxidants and zinc in the Age-Related Eye Disease Study: AREDS Report No. 13. *Archives of ophthalmology (Chicago, Ill : 1960)* 2004;122(5):716-26. doi: 10.1001/archopht.122.5.716 [published Online First: 2004/05/12]
7. Freeman EE, Egleston BL, West SK, et al. Visual acuity change and mortality in older adults. *Investigative ophthalmology & visual science* 2005;46(11):4040-5. doi: 10.1167/iovs.05-0687 [published Online First: 2005/10/27]
8. Klein R, Klein BE, Moss SE. Age-related eye disease and survival. The Beaver Dam Eye Study. *Archives of ophthalmology (Chicago, Ill : 1960)* 1995;113(3):333-9. [published Online First: 1995/03/01]
9. Lam BL, Lee DJ, Gomez-Marin O, et al. Concurrent visual and hearing impairment and risk of mortality: the National Health Interview Survey. *Archives of ophthalmology (Chicago, Ill : 1960)* 2006;124(1):95-101. doi: 10.1001/archopht.124.1.95 [published Online First: 2006/01/13]
10. Thiagarajan M, Evans JR, Smeeth L, et al. Cause-specific visual impairment and mortality: results from a population-based study of older people in the United Kingdom. *Archives of ophthalmology (Chicago, Ill : 1960)* 2005;123(10):1397-403. doi: 10.1001/archopht.123.10.1397 [published Online First: 2005/10/13]
11. Karpa MJ, Mitchell P, Beath K, et al. Direct and indirect effects of visual impairment on mortality risk in older persons. *Archives of ophthalmology (Chicago, Ill : 1960)* 2009;127(10):1347-53. doi: 10.1001/archophthalmol.2009.240 [published Online First: 2009/10/14]

For peer review only

12. Wang JJ, Mitchell P, Simpson JM, et al. Visual impairment, age-related cataract, and mortality. *Archives of ophthalmology (Chicago, Ill : 1960)* 2001;119(8):1186-90. [published Online First: 2001/08/14]
13. Mitoku K, Masaki N, Ogata Y, et al. Vision and hearing impairments, cognitive impairment and mortality among long-term care recipients: a population-based cohort study. *BMC geriatrics* 2016;16:112. doi: 10.1186/s12877-016-0286-2 [published Online First: 2016/05/29]
14. Foong AW, Fong CW, Wong TY, et al. Visual acuity and mortality in a chinese population. The Tanjong Pagar Study. *Ophthalmology* 2008;115(5):802-7. doi: 10.1016/j.ophtha.2007.04.066 [published Online First: 2007/09/04]
15. Siantar RG, Cheng CY, Gemmy Cheung CM, et al. Impact of Visual Impairment and Eye diseases on Mortality: the Singapore Malay Eye Study (SiMES). *Scientific reports* 2015;5:16304. doi: 10.1038/srep16304 [published Online First: 2015/11/10]
16. Li Z, Sun D, Liu P, et al. Visual impairment and mortality in a rural adult population (the Southern Harbin eye study). *Ophthalmic epidemiology* 2011;18(2):54-60. doi: 10.3109/09286586.2010.545503 [published Online First: 2011/03/16]
17. Khanna RC, Murthy GV, Giridhar P, et al. Cataract, visual impairment and long-term mortality in a rural cohort in India: the Andhra Pradesh Eye Disease Study. *PloS one* 2013;8(10):e78002. doi: 10.1371/journal.pone.0078002 [published Online First: 2013/11/28]
18. Cong R, Zhou B, Sun Q, et al. Smoking and the risk of age-related macular degeneration: a meta-analysis. *Ann Epidemiol* 2008;18(8):647-56. doi: 10.1016/j.annepidem.2008.04.002
19. Organisation WH. World Report on Disability. Geneva: World Health Organisation 2011.
20. Pathai S, Shiels PG, Weiss HA, et al. Ocular parameters of biological ageing in HIV-infected individuals in South Africa: relationship with chronological age and systemic biomarkers of ageing. *Mech Ageing Dev* 2013;134(9):400-6. doi: 10.1016/j.mad.2013.08.002
21. Liljas AEM, Carvalho LA, Papachristou E, et al. Self-reported vision impairment and incident prefrailty and frailty in English community-dwelling older adults: findings from a 4-year follow-up study. *J Epidemiol Community Health* 2017;71(11):1053-58. doi: 10.1136/jech-2017-209207 [published Online First: 2017/08/12]
22. Cimarolli VR, Jopp DS. Sensory impairments and their associations with functional disability in a sample of the oldest-old. *Qual Life Res* 2014;23(7):1977-84. doi: 10.1007/s11136-014-0657-0 [published Online First: 2014/04/01]
23. Bastawrous A, Mathenge W, Peto T, et al. The Nakuru eye disease cohort study: methodology & rationale. *BMC Ophthalmol* 2014;14(1):60. doi: 10.1186/1471-2415-14-60
24. Turner AG, Magnani RJ, Shuaib M. A not quite as quick but much cleaner alternative to the Expanded Programme on Immunization (EPI) Cluster Survey design. *Int J Epidemiol* 1996;25(1):198-203. [published Online First: 1996/02/01]

For peer review only

25. Kuper H, Polack S, Eusebio C, et al. A case-control study to assess the relationship between poverty and visual impairment from cataract in Kenya, the Philippines, and Bangladesh. *PLoS Med* 2008;5(12):e244.
26. Seaman SR, White IR. Review of inverse probability weighting for dealing with missing data. *Statistical methods in medical research* 2013;22(3):278-95. doi: 10.1177/0962280210395740
27. Agrawal N, Kalaivani M, Gupta SK, et al. Association of blindness and hearing impairment with mortality in a cohort of elderly persons in a rural area. *Indian journal of community medicine : official publication of Indian Association of Preventive & Social Medicine* 2011;36(3):208-12. doi: 10.4103/0970-0218.86522 [published Online First: 2011/11/18]
28. Fisher D, Li CM, Chiu MS, et al. Impairments in hearing and vision impact on mortality in older people: the AGES-Reykjavik Study. *Age and ageing* 2014;43(1):69-76. doi: 10.1093/ageing/aft122 [published Online First: 2013/09/03]
29. Kuang TM, Tsai SY, Liu CJ, et al. The association of visual impairment and 3-year mortality among the elderly in Taiwan: the Shihpai Eye Study. *Journal of the Chinese Medical Association : JCMA* 2015;78(3):177-81. doi: 10.1016/j.jcma.2014.11.006 [published Online First: 2015/01/27]
30. Mathenge W, Foster A, Kuper H. Urbanization, ethnicity and cardiovascular risk in a population in transition in Nakuru, Kenya: a population-based survey. *BMC Public Health* 2010;10:569. doi: 10.1186/1471-2458-10-569 [published Online First: 2010/09/24]
31. Knudtson MD, Klein BE, Klein R. Age-related eye disease, visual impairment, and survival: the Beaver Dam Eye Study. *Archives of ophthalmology (Chicago, Ill : 1960)* 2006;124(2):243-9. doi: 10.1001/archopht.124.2.243 [published Online First: 2006/02/16]
32. Zhu XR, Zhang YP, Bai L, et al. Prediction of risk of diabetic retinopathy for all-cause mortality, stroke and heart failure: Evidence from epidemiological observational studies. *Medicine (Baltimore)* 2017;96(3):e5894. doi: 10.1097/MD.0000000000005894
33. Cugati S, Cumming RG, Smith W, et al. Visual impairment, age-related macular degeneration, cataract, and long-term mortality: the Blue Mountains Eye Study. *Archives of ophthalmology (Chicago, Ill : 1960)* 2007;125(7):917-24. doi: 10.1001/archopht.125.7.917 [published Online First: 2007/07/11]
34. Song E, Sun H, Xu Y, et al. Age-related cataract, cataract surgery and subsequent mortality: a systematic review and meta-analysis. *PloS one* 2014;9(11):e112054. doi: 10.1371/journal.pone.0112054
35. Borger PH, van Leeuwen R, Hulsman CA, et al. Is there a direct association between age-related eye diseases and mortality? The Rotterdam Study. *Ophthalmology* 2003;110(7):1292-6. doi: 10.1016/s0161-6420(03)00450-0 [published Online First: 2003/07/18]
36. Nucci C, Cedrone C, Culasso F, et al. Association between lens opacities and mortality in the Priverno Eye Study. *Graefe's archive for clinical and experimental ophthalmology = Albrecht von Graefes Archiv fur klinische und experimentelle Ophthalmologie* 2004;242(4):289-94. doi: 10.1007/s00417-003-0837-z [published Online First: 2004/01/24]

For peer review only

37. Minassian DC, Mehra V, Johnson GJ. Mortality and cataract: findings from a population-based longitudinal study. *Bulletin of the World Health Organization* 1992;70(2):219-23. [published Online First: 1992/01/01]
38. Hennis A, Wu SY, Li X, et al. Lens opacities and mortality : the Barbados Eye Studies. *Ophthalmology* 2001;108(3):498-504. [published Online First: 2001/03/10]
39. Williams SL, Ferrigno L, Mora P, et al. Baseline cataract type and 10-year mortality in the Italian-American Case-Control Study of age-related cataract. *American journal of epidemiology* 2002;156(2):127-31. [published Online First: 2002/07/16]
40. Wong TY, Tikellis G, Sun C, et al. Age-related macular degeneration and risk of coronary heart disease: the Atherosclerosis Risk in Communities Study. *Ophthalmology* 2007;114(1):86-91. doi: 10.1016/j.ophtha.2006.06.039 [published Online First: 2007/01/03]
41. Fisher DE, Jonasson F, Eiriksdottir G, et al. Age-related macular degeneration and mortality in community-dwelling elders: the age, gene/environment susceptibility Reykjavik study. *Ophthalmology* 2015;122(2):382-90. doi: 10.1016/j.ophtha.2014.08.006 [published Online First: 2014/09/30]
42. Gopinath B, Liew G, Burlutsky G, et al. Age-related macular degeneration and risk of total and cause-specific mortality over 15 years. *Maturitas* 2016;84:63-7. doi: 10.1016/j.maturitas.2015.11.001 [published Online First: 2015/11/26]
43. Pedula KL, Coleman AL, Yu F, et al. Age-related macular degeneration and mortality in older women: the study of osteoporotic fractures. *Journal of the American Geriatrics Society* 2015;63(5):910-7. doi: 10.1111/jgs.13405 [published Online First: 2015/05/06]
44. Akbari M, Akbari S, Pasquale LR. The association of primary open-angle glaucoma with mortality: a meta-analysis of observational studies. *Archives of ophthalmology (Chicago, Ill : 1960)* 2009;127(2):204-10. doi: 10.1001/archophthalmol.2008.571
45. Boerma T. Moving towards better cause of death registration in Africa and Asia. *Glob Health Action* 2014;7:25931. doi: 10.3402/gha.v7.25931 [published Online First: 2014/11/08]
46. Reidy A, Minassian DC, Desai P, et al. Increased mortality in women with cataract: a population based follow up of the North London Eye Study. *The British journal of ophthalmology* 2002;86(4):424-8. [published Online First: 2002/03/27]
47. Lee DJ, Gomez-Marin O, Lam BL, et al. Glaucoma and survival: the National Health Interview Survey 1986-1994. *Ophthalmology* 2003;110(8):1476-83. doi: 10.1016/s0161-6420(03)00408-1 [published Online First: 2003/08/15]
48. Lee AJ, Wang JJ, Kifley A, et al. Open-angle glaucoma and cardiovascular mortality: the Blue Mountains Eye Study. *Ophthalmology* 2006;113(7):1069-76. doi: 10.1016/j.ophtha.2006.02.062 [published Online First: 2006/07/04]

For peer review only

Web Table 1. 6-Year adjusted mortality risk by level of VI among the Nakuru Eye Disease Cohort Study Participants, stratified by age and gender (Unweighted)

	Overall		Male		Female		<60 years		≥60 years	
	N	Risk per 1,000/6yrs (95%CI)	N	Risk per 1,000/6yrs (95%CI)	N	Risk per 1,000/6yrs (95%CI)	N	Risk per 1,000/6yrs (95%CI)	N	Risk per 1,000/6yrs (95%CI)
Visual acuity at baseline (better eye presenting)										
All individuals	409 / 3441	119(106,134)	236 / 1656	143(124,164)	173 / 1785	97(83,113)	86 / 1503	57(46,71)	323 / 1938	167(150,185)
Normal (≥6/12)	280 / 2901	97(84,111)	162 / 1378	118(98,140)	118 / 1523	78(64,93)	78 / 1420	55(44,69)	202 / 1481	136(118,157)
Near Normal (<6/12-≥6/18)	27 / 170	159(109,226)	14 / 84	167(104,256)	13 / 86	151(87,250)	3 / 27	111(34,309)	24 / 143	168(115,238)
VI (<6/18-≥6/60)	77 / 275	280(234,331)	45 / 142	317(252,390)	32 / 133	241(172,325)	3 / 31	97(30,268)	74 / 244	303(250,363)
SVI (<6/60-≥3/60)	4 / 16	250(83,552)	2 / 10	200(37,622)	2 / 6	333(42,851)	2 / 2	-	2 / 14	143(28,490)
Blind (<3/60)	19 / 50	380(242,541)	13 / 30	433(246,642)	6 / 20	300(129,553)	0 / 6	-	19 / 44	433(275,604)
Any VI (<6/18)	100 / 341	293(245,347)	60 / 182	330(268,398)	40 / 159	252(184,333)	5 / 39	128(52,281)	95 / 302	315(260,375)

For peer review only

STROBE 2007 (v4) Statement—Checklist of items that should be included in reports of *cohort studies*

Section/Topic	Item #	Recommendation	Reported on page #
**Title and abstract**	1	(*a*) Indicate the study's design with a commonly used term in the title or the abstract	1, 2
		(*b*) Provide in the abstract an informative and balanced summary of what was done and what was found	2
**Introduction**			
Background/rationale	2	Explain the scientific background and rationale for the investigation being reported	5
Objectives	3	State specific objectives, including any prespecified hypotheses	5
**Methods**			
Study design	4	Present key elements of study design early in the paper	6
Setting	5	Describe the setting, locations, and relevant dates, including periods of recruitment, exposure, follow-up, and data collection	6
Participants	6	(*a*) Give the eligibility criteria, and the sources and methods of selection of participants. Describe methods of follow-up	6, 7
		(*b*) For matched studies, give matching criteria and number of exposed and unexposed	-
Variables	7	Clearly define all outcomes, exposures, predictors, potential confounders, and effect modifiers. Give diagnostic criteria, if applicable	6, 7
Data sources/ measurement	8*	For each variable of interest, give sources of data and details of methods of assessment (measurement). Describe comparability of assessment methods if there is more than one group	7
Bias	9	Describe any efforts to address potential sources of bias	8
Study size	10	Explain how the study size was arrived at	6
Quantitative variables	11	Explain how quantitative variables were handled in the analyses. If applicable, describe which groupings were chosen and why	7,8
Statistical methods	12	(*a*) Describe all statistical methods, including those used to control for confounding	7,8
		(*b*) Describe any methods used to examine subgroups and interactions	7,8
		(*c*) Explain how missing data were addressed	7,8
		(*d*) If applicable, explain how loss to follow-up was addressed	7,8
		(*e*) Describe any sensitivity analyses	7,8
**Results**			

For peer review only

Participants	13*	(a) Report numbers of individuals at each stage of study—eg numbers potentially eligible, examined for eligibility, confirmed eligible, included in the study, completing follow-up, and analysed	10
		(b) Give reasons for non-participation at each stage	10
		(c) Consider use of a flow diagram	-
Descriptive data	14*	(a) Give characteristics of study participants (eg demographic, clinical, social) and information on exposures and potential confounders	10
		(b) Indicate number of participants with missing data for each variable of interest	In tables
		(c) Summarise follow-up time (eg, average and total amount)	10
Outcome data	15*	Report numbers of outcome events or summary measures over time	10
Main results	16	(*a*) Give unadjusted estimates and, if applicable, confounder-adjusted estimates and their precision (eg, 95% confidence interval). Make clear which confounders were adjusted for and why they were included	Tables 1 and 2
		(*b*) Report category boundaries when continuous variables were categorized	All tables
		(*c*) If relevant, consider translating estimates of relative risk into absolute risk for a meaningful time period	-
Other analyses	17	Report other analyses done—eg analyses of subgroups and interactions, and sensitivity analyses	10
**Discussion**			
Key results	18	Summarise key results with reference to study objectives	12
**Limitations**			
Interpretation	20	Give a cautious overall interpretation of results considering objectives, limitations, multiplicity of analyses, results from similar studies, and other relevant evidence	13
Generalisability	21	Discuss the generalisability (external validity) of the study results	12
**Other information**			
Funding	22	Give the source of funding and the role of the funders for the present study and, if applicable, for the original study on which the present article is based	3

*Give information separately for cases and controls in case-control studies and, if applicable, for exposed and unexposed groups in cohort and cross-sectional studies.

Note: An Explanation and Elaboration article discusses each checklist item and gives methodological background and published examples of transparent reporting. The STROBE checklist is best used in conjunction with this article (freely available on the Web sites of PLoS Medicine at http://www.plosmedicine.org/, Annals of Internal Medicine at http://www.annals.org/, and Epidemiology at http://www.epidem.com/). Information on the STROBE Initiative is available at www.strobe-statement.org.

For peer review only

BMJ OPEN response

1. The Nakuru Eye Disease Cohort Study was described as a two-stage cluster sampling with "a probability proportional to the size of the population" for the selection of clusters and "a modified compact segment sampling method" for the selection of households within clusters. However, the authors responded that "the original survey was a self-weighting sample, so no adjustments were made for sampling probability". This appeared to be contradictory to each other. Similarly, the authors stated that "All analyses accounted for the cluster survey design using Taylor linearized variance estimation to calculate standard errors", which again was contradictory to the statement "no adjustments were made for sampling probability". Therefore, the authors need to clarify whether their analyses were corrected for the cluster survey design only or IPW model only or both.

Response: The analyses corrected for the cluster survey design and IPW model, which was already mentioned in the text, but has now been further clarified (page 8, last paragraph).

If the authors controlled for both, please describe how they did this.

Response: Control for both was undertaken by using svyset command in Stata with cluster as the primary sampling unit and weighting using the inverse of the probability of being followed up. This has now been clarified in the text (page 8, last paragraph).

If they only corrected for one, please be aware that the study sample was not a random independent (and representative) sample and there would be limitations to the generalizability of the study results and conclusions (and this would no longer be a strength of this study).

Response: We corrected for both.

Please also note that the development of the IPW model should be corrected for the cluster survey design too as the study sample was not a random independent sample.

Response: Thank you for the comment, the IPW model was corrected for the cluster survey design too and this has been clarified in the text (page 8, last paragraph).

It will be helpful if the authors can provide the final covariates included in the IPW model.

Response: The covariates were age, sex, rural/urban, mother tongue. This has been clarified in the text (Page 8, paragraph 3).

2. The authors mentioned that the overall mortality was estimated by assuming a Poisson distribution of events for the population overall and stratified by each covariate. However, it is not clear what is the unit for adjusted Poisson regression models. Did the authors analyze the mortality status for each subject as the outcome and assume this binary outcome following a Poisson distribution?

For peer review only

Response: Mortality status was the binary outcome and the distribution was assumed to be Poisson. This has been clarified in the text (page 8, last paragraph).

Also, the duration of follow-up was not the same for each subject, so the assumption of same duration of follow-up for all study participants was questionable. Since the duration of follow-up was not taken into account in the analysis and the unit of analysis was subject, what was the rationale for the choice of Poisson regression models versus logistic regression models?

Response: The study participants were only contacted at two time points and so it was not possible to estimate the follow-up for each individual participant. Poisson regression models were chosen rather than logistic regression as these generate risk ratios instead of odds ratios and are therefore easier to interpret.

3. As the date of death was not available in this study, the mean follow-up of 5.6 year must only be calculated among 2,170 subjects who were re-examined, not among all 3,441 subjects who were included in this study (or all 4,414 subjects who were examined at baseline). If so, please state this clearly in the paper. In addition, if this was true, the overall mean follow-up for the total study population should be much shorter, and I don't think that it is appropriate to state 6-year mortality risk as the outcome for this study. It is also inappropriate to use the term simply due to its use in other publications as it is clearly not accurate in this study. For example, if the authors will conduct another follow-up examination in this cohort with only 10% subjects who will be re-examined and they calculate the mean follow-up as 15 years among these 10% subjects, will they still call such a future study as 15-year mortality based on the mean follow-up of 10% subjects when they know 90% subjects have <15 years of follow-up?

Response: The mean follow-up refers to the time between baseline and follow-up, not the average time that each individual is followed-up in the cohort. This has been clarified in the text (Page 10, paragraph 1).

4. Were results in Table 3 based on the Poisson regression models? If so, the estimates should be RR not OR.

Response: We have corrected these to read RR, not OR (Table 3).

For peer review only

Mortality during six years of follow-up in relation to visual impairment and eye disease: Results from a population-based cohort study of people aged 50 years and above in Nakuru, Kenya

Journal:	*BMJ Open*
Manuscript ID	bmjopen-2019-029700.R1
Article Type:	Research
Date Submitted by the Author:	04-Apr-2019
Complete List of Authors:	Kuper, Hannah; The London School of Hygiene & Tropical Medicine, Clinical Research<br>Mathenge, Wanjiku; Univ London London Sch Hyg<br>Macleod, David; Univ London London Sch Hyg<br>Foster, Allen; Univ London London Sch Hyg<br>Gichangi, Michael; Division of Preventive Ophthalmic Services, Ministry of Public Health and Sanitation<br>Rono, Hillary; Univ London London Sch Hyg<br>Wing, Kevin; London School of Hygiene and Tropical Medicine,<br>Weiss, Helen; London School of Hygiene and Tropical Medicine,<br>Burton, Matthew J; London School of Hygiene and Tropical Medicine<br>Bastawrous, Andrew; Univ London London Sch Hyg ,
**Primary Subject Heading**:	Epidemiology
Secondary Subject Heading:	Epidemiology, Ophthalmology
Keywords:	blindness, Kenya, mortality

SCHOLARONE™
Manuscripts

Mortality during six years of follow-up in relation to visual impairment and eye disease: Results from a population-based cohort study of people aged 50 years and above in Nakuru, Kenya

Authors: Hannah Kuper, ScD[1,2]*, Wanjiku Mathenge, PhD[3], David Macleod, MSc[4], Allen Foster, FRCS[1,2], Michael Gichangi, MSc[5], Hillary Rono, MSc[1,6], Kevin Wing, PhD[7], Helen A Weiss, DPhil[4], Matthew Burton, PhD[1], Andrew Bastawrous, PhD[1]

*Corresponding author: Professor Hannah Kuper, London School of Hygiene and Tropical Medicine, London, United Kingdom. E-mail: Hannah.kuper@lshtm.ac.uk

[1]International Centre for Eye Health, Clinical Research Department, London School of Hygiene and Tropical Medicine, London, United Kingdom

[2]International Centre for Evidence in Disability, Clinical Research Department, London School of Hygiene and Tropical Medicine, London, United Kingdom

[3]Rwanda International Institute of Ophthalmology and Dr. Agarwal's Eye Hospital, Kigali, Rwanda

[4]MRC Tropical Epidemiology Group, Department of Infectious Disease Epidemiology, London School of Hygiene & Tropical Medicine, London, United Kingdom

[5]Ministry of Health, Nairobi, Kenya

[6]Kitale Eye Unit, Ministry of Health Trans Nzoia County, Kenya

[7] Department of Non-Communicable Disease Epidemiology, London School of Hygiene & Tropical Medicine, London, United Kingdom

Manuscript word count: 3139

For peer review only

Abstract

Objective: To estimate the association between (1) visual impairment (VI) and (2) eye disease and 6-year mortality risk within a cohort of elderly Kenyan people.

Design, setting and participants: The baseline of the Nakuru Posterior Segment Eye Disease Study was formed from a population-based survey of 4318 participants aged ≥50 years, enrolled in 2007-2008. Ophthalmic and anthropometric examinations were undertaken on all participants at baseline, and a questionnaire was administered, including medical and ophthalmic history. Participants were retraced in 2013-2014 for a second examination. Vital status was recorded for all participants through information from community members. Cumulative incidence of mortality, and its relationship with baseline VI and types of eye disease was estimated. Inverse probability weighting was used to adjust for nonparticipation.

Primary outcome measures: Cumulative incidence of mortality in relation to VI level at baseline.

Results: Of the baseline sample, 2,170 (50%) were re-examined at follow-up and 407 (10%) were known to have died (adjusted risk of 11.9% over 6 years). Compared to those with normal vision (visual acuity (VA) ≥6/12, risk=9.7%), the 6-year mortality risk was higher among people with VI (<6/18-≥6/60; risk=28.3%; RR=1.75, 1.28-2.40) or severe VI/blindness (<6/60; risk=34.9%; RR=1.98, 1.04-3.80). These associations remained after adjustment for Non Communicable Disease (NCD) risk factors (mortality: RR=1.56, 95% CI 1.14-2.15; SVI/blind: RR=1.46, 95% CI 0.80-2.68). Mortality risk was also associated with presence of diabetic retinopathy at baseline (RR=3.18, 95% CI=1.98-5.09), cataract (RR=1.26, 0.95-1.66), and presence of both cataract and VI (RR=1.57, 1.24-1.98). Mortality risk was higher among people with age-related macular degeneration at baseline (with or without VI), compared with those without (RR=1.42, 0.91-2.22 and RR=1.34, 0.99-1.81, respectively).

Conclusions: Visual acuity was related to six-year mortality risk in this cohort of elderly Kenyan people, potentially because both VI and mortality are related to ageing and risk factors for NCD.

For peer review only - http://bmjopen.bmj.com/site/about/guidelines.xhtml

Strengths and limitations of this study

- The cohort comprised of a representative population-based sample in an area of ethnic, socioeconomic, and educational diversity.
- There was comprehensive assessment of ophthalmic characteristics and risk factors at baseline and follow-up.
- Data on mortality was collected through informant report, rather than from death certificates.
- There was a high loss to follow-up in this study, raising the possibility of selection bias.

Funding/Support: This study was supported by grant G1001934 from the Medical Research Council, grant 1310 from Fight for Sight, the British Council for the Prevention of Blindness, and the International Glaucoma Association (Dr Bastawrous). Dr Burton is supported by grant 098481/Z/12/Z from the Wellcome Trust. Dr Weiss is supported by grant G0700837 from the Medical Research Council and Department for International Development. The funding sources had no role in the design and conduct of the study; collection, management, analysis, and interpretation of the data; preparation, review, or approval of the manuscript; and the decision to submit the manuscript for publication.

Author Contributions: Professor Kuper had full access to all the data in the study and takes responsibility for the integrity of the data and the accuracy of the data analysis.

- *Study concept and design:* Bastawrous, Mathenge, Foster, Burton, Kuper.
- *Acquisition, analysis, or interpretation of data:* Bastawrous, Wing, Rono, Weiss, Macleod, Burton.
- *Drafting of the manuscript:* Kuper, Bastawrous, Macleod.
- *Critical revision of the manuscript for important intellectual content:* Bastawrous, Mathenge, Gichangi, Wing, Rono, Weiss, Foster, Burton, Kuper.
- *Statistical analysis:* Kuper, Bastawrous, Wing, Weiss, Macleod.
- *Obtained funding:* Bastawrous, Kuper.

For peer review only

- *Administrative, technical, or material support:* Bastawrous, Mathenge, Gichangi, Rono, Kuper.
- *Study supervision:* Bastawrous, Foster, Burton, Kuper.

Conflict of Interest: None.

Data sharing: Data is available on request from Andrew Bastawrous or Hannah Kuper.

For peer review only

Introduction

Visual impairment (VI) is common, affecting approximately 253 million people globally. [1] It can impact on different aspects of people's lives, including reducing quality of life, and increasing poverty and depression. [2-5] There is growing evidence from Europe, North America, Asia and Australia that VI and specific eye conditions are linked to increased risk of mortality,[6-17] but data are lacking for Low and Middle Income Countries (LMIC), particularly from Sub-Saharan Africa.

There are several potential pathways by which VI may be linked with mortality. Both VI and mortality are related to ageing, and so confounding or residual confounding may explain the reported associations. There are also common underlying risk factors for both VI and mortality, such as smoking, obesity, and poverty. For instance, VI due to age-related macular degeneration (AMD) is more common among smokers, [18] and smoking increases risk of mortality. An underlying disease may also cause both VI and mortality, for instance diabetic retinopathy (DR) is related to poor control of diabetes, which also causes increased mortality. People with VI may find it more difficult to seek health-care, due to a range of barriers,[19] thereby increasing their mortality risk. Changes in the eye may be a marker of ageing, or accelerated ageing,[20] and thereby linked to mortality. Finally, VI could acerbate frailty, depression and functional difficulties, all linked to increased mortality. [3 21 22]

It is important to explore whether there is an association between VI and mortality and, if there is, to identify possible pathways for this link, in order to understand how to reduce the vulnerability of people with VI to increased morbidity and mortality. Furthermore, these data may be useful to advocate for scaling up of eye health services in LMICs. The objective of this study was to investigate the association between VI and six-year mortality risk within the Nakuru Eye Disease Cohort Study, a cohort of elderly Kenyan people.

For peer review only

For peer review only - http://bmjopen.bmj.com/site/about/guidelines.xhtml

Materials and Methods

The methodology of the Nakuru Eye Disease Cohort Study has been reported previously,[23] and is summarised here.

Baseline Study Population

The baseline population based survey was conducted in 2007/8. A total of 100 clusters each of 50 participants were selected with a probability proportional to the size of the population across Nakuru district. Households were selected within clusters using a modified compact segment sampling method.[24] Eligible individuals were those aged ≥50 years living in the household for at least three months in the previous year, and multiple subjects could be included per household.

Baseline Ophthalmic and General Examination

All participants were invited to undergo a comprehensive ophthalmic examination at a screening clinic. [23] The objectives of the survey and the examination process were explained to those eligible in the local dialect, in the presence of a witness. A subject was examined only after informed written (or thumbprint) consent was obtained.

All participants underwent logMAR visual acuity testing on each eye separately and corrected visual acuity (by refraction or pinhole) when less than 20/40 Snellen equivalent. Participants had 2 non-stereoscopic, digital, 45° fundus photographs (1 disc and 1 macula centered) taken per eye by an ophthalmic clinical officer. Digital images were graded for the presence of AMD and DR at an approved grading center (Moorfields Eye Hospital Reading Centre) by a senior grader, with adjudication by a clinician for confirmed cases and 5% of randomly selected images to ensure quality control. The presence of cataract was recorded by the ophthalmologist (WM) on slit-lamp examination after pupil dilation.

Detailed interviews were undertaken in the local language on demographic details, information on risk factors, socioeconomic status (SES), and full medical history.

A nurse performed and recorded measures of participants: height (Leicester Height Measure); weight (Seca 761); and three measures of blood pressure (Omron® Digital Automatic Blood Pressure Monitor Model HEM907), each ten minutes apart. Capillary blood was taken from all participants for random blood glucose (Accutrend GC system).

For peer review only - http://bmjopen.bmj.com/site/about/guidelines.xhtml

Assessment of vital status at follow-up

Follow-up was conducted from January 2013 to March 2014. A meeting was held approximately one week before the follow-up examination clinic for a given cluster. A list of study participants was given to the chief and a local village guide was recruited to assist location of the study participants. The village guide was someone who knew and was well known by the community (or the village chief him/herself). The Advance Team visited homes of baseline participants on the day prior to the examination clinic and confirmed their identity using National Identity cards and invited them to attend the examination clinic the following day. All identified participants were also asked to help locate baseline participants that had not been found.

Each local field guide was asked to classify the baseline study participant for that cluster as "available", "died", "moved away" or "unknown". A participant was defined to have died if this was verified by at least two people from amongst the village chief, local guide or available study participant. Those who were known to have moved away were contacted when possible to either arrange follow-up at a more suitable location for the participant or to identify if they were alive or had died in the follow-up period. Any participant for whom nobody could identify as being alive or having moved away was recorded as "unknown".

Definitions and Statistical Analyses

All participants who had complete examinations at baseline were considered "at-risk" for mortality during follow up. Follow-up status at 6 years was categorised as:

i) Deceased (confirmed dead, as described above);

ii) Alive (i.e. re-examined at follow-up, retraced but refused or unavailable at follow-up, or moved away but known to be alive)

iii) Unknown (i.e. not retraced at follow-up, death not verified as described above, or moved away but vital status unknown).

A socioeconomic status (SES) score was developed based on information collected on job, housing conditions, and ownership of material goods and livestock, based on previous work in the same population. [25] Hypertension was defined on the basis of the average of the second and third reading, with cut-offs used of systolic blood pressure≥140 mmHG and/or diastolic blood

For peer review only - http://bmjopen.bmj.com/site/about/guidelines.xhtml

pressure≥90 mmHG and/or self-reported hypertension medication. Diabetes was defined as (1) Self-reported in the history, or (2) random glucose of ≥11.0mmol/L.

Statistical analysis was performed using STATA v14 (Stata Corp). All analyses accounted for the cluster survey design using Taylor linearized variance estimation to calculate standard errors. Pearson Chi-squared tests corrected for the survey-design were used to calculate p-values to assess differences between participants whose mortality status is known and those where mortality status is unknown, i.e. lost to follow-up (LTFU).

An inverse probability-weighting (IPW) model[26] was developed, in order to allow estimation of mortality risk while accounting for those LTFU. Multivariable logistic regression was used to identify independent baseline covariates associated with LTFU. Covariates for which there was evidence of univariable association with the outcome ($p<0.10$ across all categories of the variable) were kept in a multivariable model (age, sex, rural/urban and mother tongue). From this final model, the probability of being followed up was estimated, based on the presence or absence of each of these baseline covariates. The inverse of this probability formed the weighting to be applied to account for those LTFU.

The final step was to remove those individuals LTFU from the cohort, so that all subsequent analysis would be performed on only those with complete outcome records, with IPW applied to account for those LTFU. A sensitivity analyses for this approach involved repeating the analyses without applying IPW (i.e. standard unweighted complete case analysis), and assessing the impact on the results.

Six-year mortality risk was calculated by dividing the number of deaths identified at follow-up by the number of people at risk at baseline. 95% confidence intervals were estimated assuming a Poisson distribution of events. This was done for the population overall, and stratified by each covariate.

Age/sex-adjusted risk ratios for each covariate in relation to mortality were estimated using a Poisson regression model with robust error variance to allow for the clustered design and including IPW. Mortality status was the binary outcome and the distribution was assumed to be Poisson. These analyses were adjusted for the clustered design, as well as the use of IPW, by setting the clusters as the primary sampling unit and weighting using the inverse probability of being followed-up. The model was further adjusted using a set of four socioeconomic (SES) variables only (SES quartile, location, ethnic group, education), then a set of five non-

For peer review only - http://bmjopen.bmj.com/site/about/guidelines.xhtml

communicable disease (NCD) risk factors only (smoking, alcohol, diabetes, hypertension, BMI) before finally adjusting for all nine SES and NCD variables.

Patient and Public Involvement

Patients and the public were not directly involvement in the development of the research question and outcome measures, or the design or conduct of study. There are no plans to disseminate results directly to the study participants.

Ethical Approval

The study adhered to the tenets of the Declaration of Helsinki and was approved by the Ethics Committee of London School of Hygiene and Tropical Medicine at both baseline and follow-up (LSHTM Ref 6192). Baseline approval was provided by the Kenya Medical Research Institute Ethics Committee and by the African Medical and Research Foundation (AMREF) Ethics Committee, Kenya for the follow-up (AMREF-ESRC P44/12). For both phases, approval was granted by the Rift Valley Provincial Medical Officer and the Nakuru District Medical Officer of Health. Approval was sought from the administrative heads in each cluster.

For peer review only

Results

At baseline 4,414 participants were examined. The follow-up assessment was conducted, on average, 5.6 years (SD 0.6) after the baseline, expressed for simplicity as 6 years (meaning that there was 6 years between the baseline and follow-up wave, rather than that each participant was followed-up on average for 6 years as the time of loss to follow-up or death was not known for individuals). Of the baseline participants, 3032 were known to be alive at follow-up (69% 2,170 re-examined at the follow-up plus 862 known to be alive but not re-examined), 409 (9%) were known to have died, and 973 had unknown vital status (22%)..

Table 1 provides the baseline characteristics of participants who had died during the follow-up, those were re-examined at follow-up and those who were LTFU. In comparison to those who had died, those who were re-examined were younger, more likely to be female, Kalenjin speakers, and had higher SES, while those of unknown status were more likely to be of "other" tribes and urban residence.

Table 2 shows the weighted 6-year mortality risk by level of VI. Overall, the 6-year mortality risk was 11.9% over 6 years. Risk increased with worsening levels of VI, from 9.7% (95% CI 8.4%-11.1%) among those with normal vision to 38.5% (24.5-54.8%) among those who were blind. This pattern was observed in both males and females, but was less clear in people aged <60 years given the low mortality in this group and consequent small numbers. In each sub-group, the lowest risk of mortality was among people with normal vision. Mortality risk among people with VI was higher for males than for females, and among those ≥60 years versus <60 years. Estimates changed little after weighting for LTFU (web table for unweighted estimates).

Compared to those with normal vision (VA>6/12, risk=9.7%), the mortality risk was significantly higher among people with VI (VA<6/18-≥6/60; risk=28.3%; RR=1.75, 95% CI 1.28-2.40) or SVI/blindness (VA<6/60: risk=34.9%; RR=1.98, 1.04-3.80) (Table 3). There was a weakening of the association after adjustment for non-communicable disease (NCD) risk factors or full adjustment for both socio-economic status (SES) and NCD risk factors, although the overall trends between worsening vision and increased 6-year mortality risk remained evident (VI: RR=1.56, 95% CI 1.14-2.15; SVI/blind: RR=1.46, 95%CI 0.80-2.68).

People with any VI had a higher mortality risk than those without VI (29.7% versus 9.7%; RR=1.54, 95%CI 1.22-1.93), and this association remained after adjustment for SES and NCD risk factors (RR=1.37, 95%CI 1.10-1.71) (Table 4). Other risk factors associated with 6-year mortality risk after comprehensive adjustment included increasing age (oldest versus youngest age group: RR=4.68, 95%CI 3.55-6.18) and diabetes (RR=2.34, 95%CI 1.81-3.03). Being underweight was associated with an increased 6-year mortality risk (underweight

For peer review only - http://bmjopen.bmj.com/site/about/guidelines.xhtml

versus normal: RR=1.60, 95%CI 1.24-2.07).

Risk of mortality was analysed by prevalence of specific eye diseases at baseline (Table 5). The presence of cataract (or aphakia/pseudophakia) and any VI (i.e. VA <6/18 in better eye) was associated with higher mortality risk (RR=1.57, 95%CI 1.24-1.98), whereas cataract alone (or aphakia/pseudophakia) was not (RR=1.26, 95%CI 0.95-1.66). Mortality risk was higher among people with AMD at baseline (with or without VI), compared with those without, although these associations were not statistically significant (RR=1.42, 95%CI 0.91-2.22 and RR=1.34, 95%CI 0.99-1.81, respectively). DR was associated with a three-fold increased mortality risk (RR=3.18, 95%CI 1.98-5.09). The number of people with DR and any VI were too small to make meaningful inferences.

For peer review only

For peer review only - http://bmjopen.bmj.com/site/about/guidelines.xhtml

Discussion

VI was associated with increased mortality risk during six years of follow-up in a cohort of elderly Kenyan people. The risk of mortality increased with worsening vision. This association was reduced after adjustment for the presence of NCD risk factors, and to a lesser extent for SES indicators. Among eye conditions, DR was most strongly associated with mortality risk, although the number affected was small. Cataract with VI was also associated with elevated mortality, as were AMD and cataract without visual loss at baseline (although these estimates lacked precision).

Previous studies have also shown a positive relationship between VI and mortality, with evidence available from the USA [6-9], UK[10], Australia[11 12], Japan [13], Singapore [14 15], China[16] and India [17]. Others have failed to find evidence for this association, including in India [27], Iceland [28], and Taiwan. [29] Data from LMICs are sparse, in particular for Sub-Saharan Africa, and so comparison of our study findings to those from similar settings is not possible.

On the basis of our findings and those in the wider literature consideration can be given to the potential pathway for the association between VI and mortality. There was clear evidence for confounding by age, as both VI and mortality are independently related to older age. Consequently, imperfect adjustment for age may have allowed for residual confounding as a partial explanation for the association. There was little evidence for confounding by SES, although in this setting high SES was associated with greater prevalence of NCD risk factors,[30] and a somewhat reduced mortality risk. The presence of NCD risk factors may also act as confounders of the association of VI on mortality, since the association was attenuated after adjustment for these indicators, as found in other studies.[10] Significant associations persisted, however, between VI and mortality after comprehensive multivariable adjustment in this study, as occurred in previous studies, [12-14 17 31] suggesting that residual confounding or direct effects of VI on mortality may be operational.

Exploring the relationship between different eye conditions and mortality may help to clarify whether independent biological pathways exist. DR is known to be associated with increased mortality,[15 32] as was also shown in this study. This link is unsurprising given the well-known relationship between uncontrolled diabetes with both DR and mortality. However, the relatively small number of people with DR in this population means that this link cannot be the

For peer review only - http://bmjopen.bmj.com/site/about/guidelines.xhtml

sole driver of the VI-mortality association. Our study as well as others have shown cataract to be associated with increased mortality. [6 12 17 33 34], though this association is not always demonstrated.[15 35] Some studies have suggested that this relationship varies by cataract type. [8 34 36-38] It is hypothesized that the association between cataract and mortality arises as lens opacification (cataract) is an indicator of accelerated ageing. [20 39] The evidence for a link between AMD and mortality is more complex; some studies show that late AMD is associated with mortality, but not early AMD. [6 40-42] Others found no association between AMD and mortality, [15 33 35] or only among women.[43]

There are other potential pathways between VI and mortality not explored in this study. For example, NCD risk factors may be mediators of the effect of VI on mortality (rather than confounders) for reasons such as lower ability to access NCD treatment, less exercise, poorer diets and so on. Consequently, the association between VI and mortality adjusted by NCD risk factors would be an underestimate of the total effect. There were also concerns about the accuracy of assessment of visual fields in this population. Consequently it was not possible to determine the presence of glaucoma at baseline, although others have suggested a link between glaucoma and mortality. [15 44] We also did not assess the impact of VI in accessing health care, although the Australian Blue mountain study showed that difficulties in walking explained some of the link between VI and mortality.[11] Only 18 people with cataract underwent cataract surgery during the follow-up period, so it was not possible to assess the impact on mortality.

There are several further limitations of the study, which need to be considered when interpreting the findings. There was a lack of data on date of death, and no verification from death certificates, as these are rarely available in many African settings, [45] including Kenya. Cause of death could not be determined, and so we could not assess whether the relationship was stronger between VI and specific causes of mortality, notably cardiovascular and non-cancer causes, as demonstrated in previous studies,[15 46-48] which would lend weight to a biological pathway for the association. The follow-up study was conducted after a period of post-election violence in the area. Consequently, there was a high loss to follow-up in this study, raising the possibility of selection bias influencing the findings, although patterns changed little after weighting for loss to follow up. We did not adjust for the population sampling weights in our analysis, and so there could be concerns about the representativeness of the sample, although the selected sample had a similar demographic distribution to the general population. [49] Furthermore, the mortality rate may have been higher in this period, due to violence, and may have biased the association with VI if these deaths were disproportionally among people with VI, or among younger people (with lower prevalence of VI). Date of loss

For peer review only

to follow-up or death was not recorded, and so survival analysis was not possible. Another concern is that reports of local informants was used to categorise some people who had moved away as “known to be alive”, which may have created inaccuracies. The study may have been under-powered for some of the sub-group analyses, such as assessing the link between type of eye disease and mortality. We did not evaluate the association of different sub-types of AMD or cataract in relation to mortality because of small numbers.VI classification did not include loss of visual fields, and so the prevalence of functionally significant sight loss may have been underestimated. Self-reported diabetes was not confirmed (e.g. from medical records). In terms of strengths, this was the first study of its kind in sub-Saharan Africa to assess the association between VI and mortality. The study participants comprised a representative population-based sample in an area of ethnic, socioeconomic, and educational diversity. There was comprehensive assessment of ophthalmic characteristics and risk factors at baseline, and every attempt was made to follow up all participants, and to record vital status.

In conclusion, visual acuity was related to six-year mortality risk in this cohort of elderly Kenyan people. The most likely explanation for the association is that both VI and mortality are related to ageing and NCD risk factors. The implication is that continuity of care is needed, as people with VI require linkages to preventative and treatment services. Furthermore, we must advocate for the scale-up of eye care services in Kenya, as VI is linked to premature mortality.

For peer review only

Table 1. Baseline Characteristics of the Nakuru Eye Disease Cohort Study

**Baseline Characteristics**		*Deceased N=409*	*Followed-up or known to be alive n=3,032*	*p-value**	*Unknown status N=973*	*p-value ** *
Age in years, mean (SD)		71.6 (12.8)	62.4 (9.6)	<0.001	63.2 (10.6)	0.60
Sex, % (n)	Female	42% (173)	53% (1,612)	<0.001	53% (516)	0.59
	Male	58% (236)	47% (1,420)		47% (457)	
Tribe, % (n)	Kikuyu	69%(283)	62% (1,881)	0.02	61% (596)	0.005
	Kalenjin	23% (93)	24% (736)		19% (186)	
	Other	8% (33)	14% (415)		20% (191)	
Education, % (n)***	None	6% (26)	9% (283)	<0.001	12% (114)	0.21
	Primary	44% (179)	32% (952)		33% (323)	
	Secondary	43% (174)	48% (1,448)		44% (427)	
	Higher	7% (29)	11% (327)		11% (105)	
Residence, % (n)	Rural	74% (303)	71% (2,167)	0.43	51% (498)	<0.001
	Urban	26% (106)	29% (865)		49% (475)	
SES Quartile, % (n)***	Lower	33% (136)	24% (709)	0.002	26% (247)	0.17
	Middle lower	24% (96)	26% (776)		23% (219)	
	Middle upper	24% (97)	26% (777)		23% (218)	
	Upper	19% (79)	24% (731)		29% (281)	

*p-value describes the strength of evidence that each variable is associated with mortality, among those where we know the mortality status (Null hypothesis is

For peer review only - http://bmjopen.bmj.com/site/about/guidelines.xhtml

that the odds of death are equal in each category of the variable)

**p-value describes the strength of evidence that each variable is associated with mortality status being missing (i.e. comparison of known versus unknown mortality status). (Null hypothesis is that odds of knowing the mortality status of an individual at follow up are equal in each group)

*** There were 27 missing values for education, and 48 missing values for SES.

For peer review only

For peer review only - http://bmjopen.bmj.com/site/about/guidelines.xhtml

Table 2. 6-Year weighted mortality risk by level of VI among the Nakuru Eye Disease Cohort Study Participants, stratified by age and gender

WEIGHTED USING IPWs	Overall		Male		Female		<60 years		≥60 years	
	N	Risk per 1,000/6yrs (95%CI)	N	Risk per 1,000/6yrs (95%CI)	N	Risk per 1,000/6yrs (95%CI)	N	Risk per 1,000/6yrs (95%CI)	N	Risk per 1,000/6yrs (95%CI)
Visual acuity at baseline (better eye presenting)										
All individuals	409 / 3441	119(106,134)	236 / 1656	143(123,165)	173 / 1785	98(84,113)	86 / 1503	56(45,70)	323 / 1938	169(151,188)
Normal (≥6/12)	280 / 2901	97(84,111)	162 / 1378	118(98,142)	118 / 1523	77(64,93)	78 / 1420	54(42,68)	202 / 1481	138(120,159)
Near Normal (<6/12-≥6/18)	27 / 170	158(107,226)	14 / 84	168(104,258)	13 / 86	148(85,245)	3 / 27	106(32,301)	24 / 143	168(114,241)
VI (<6/18-≥6/60)	77 / 275	283(236,336)	45 / 142	316(252,388)	32 / 133	249(178,338)	3 / 31	99(31,271)	74 / 244	307(252,368)
SVI (<6/60-≥3/60)	4 / 16	260(86,566)	2 / 10	187(34,603)	2 / 6	379(51,875)	2 / 2	-	2 / 14	136(27,475)
Blind (<3/60)	19 / 50	385(245,548)	13 / 30	436(247,646)	6 / 20	310(133,570)	0 / 6	-	19 / 44	438(279,612)
Any VI (<6/18)	100 / 341	297(248,351)	60 / 182	328(268,395)	40 / 159	262(191,347)	5 / 39	137(57,297)	95 / 302	318(262,380)

For peer review only

Table 3: Association between visual acuity category and 6-year mortality risk

VA category	Age-sex adjusted RR	Age-sex, SES* adjusted RR	Age-sex, NCD risk** factor adjusted RR	Fully adjusted*** RR
Normal (≥6/12)	Reference	Reference	Reference	Reference
Near Normal (<6/12-≥6/18)	0.92 (0.57 – 1.50)	0.84 (0.51 – 1.39)	0.87 (0.51 – 1.48)	0.82 (0.48 – 1.41)
VI (<6/18-≥6/60)	1.75 (1.28 – 2.40)	1.77 (1.30 – 2.40)	1.56 (1.13 – 2.16)	1.56 (1.14 – 2.15)
SVI/blind (<6/60)	1.98 (1.04 – 3.80)	1.95 (1.01 – 3.76)	1.51 (0.82 – 2.77)	1.46 (0.80 – 2.68)
p-value	0.004	0.003	0.04	0.03

*SES = SES quartile, location, ethnic group, education

**NCD risk factor = smoking, alcohol, diabetes, hypertension, BMI

***Age-sex, plus all SES and NCD risk factors

For peer review only

Table 4. Multivariable analysis of baseline co-variables and 6-year mortality risk in the Nakuru Eye Disease Cohort Study

	No at risk	Deaths	Risk per 1,000/6yrs (95%CI)	Age-sex-adjusted Risk Ratio	Age-sex-SES adjusted Risk Ratio	Age-Sex-SES-NCD risk factor adjusted Risk Ratio
Any VI (<6/18)						
- No	3071	307	100(87,115)	Reference	Reference	Reference
- Yes	341	100	297(248,351)	1.54(1.22,1.93)	1.55(1.24,1.94)	1.37(1.10,1.71)
Gender						
- Male	1656	236	143(123,165)	Reference	Reference	Reference
- Female	1785	173	98(84,113)	0.74(0.63,0.87)	0.68(0.56,0.83)	0.82(0.63,1.06)
Age						
- 50-59	1503	86	56(45,70)	Reference	Reference	Reference
- 60-69	1036	96	94(77,115)	1.64(1.24,2.17)	1.58(1.19,2.09)	1.40(1.07,1.83)
- 70-79	571	107	191(161,224)	3.27(2.55,4.20)	3.15(2.39,4.15)	2.74(2.09,3.60)
- 80+	331	120	363(311,419)	6.36(4.85,8.33)	5.76(4.39,7.57)	4.68(3.55,6.18)
**SES risk factors**						
Location						
- Rural	2470	303	123(108,140)	Reference	Reference	Reference
- Urban	971	106	110(83,145)	1.14(0.90,1.45)	1.15(0.90,1.48)	1.18(0.91,1.53)
SES quartile						
- Lower	845	136	164(137,197)	Reference	Reference	Reference

For peer review only

- Lower middle	872	96	110(91,134)	0.75(0.60,0.93)	0.72(0.58,0.91)	0.75(0.59,0.95)
- Upper middle	874	97	110(89,134)	0.86(0.66,1.11)	0.82(0.63,1.06)	0.84(0.65,1.09)
- Upper	810	79	99(78,126)	0.89(0.69,1.14)	0.78(0.58,1.05)	0.76(0.56,1.03)
Ethnic group						
- Kikuyu	2164	283	131(116,148)	Reference	Reference	Reference
- Kalenjin	829	93	113(91,139)	0.83(0.66,1.03)	0.78(0.62,0.99)	0.81(0.63,1.04)
- Other	448	33	76(48,119)	0.84(0.56,1.26)	0.82(0.55,1.21)	0.81(0.55,1.20)
Education						
- No education	309	26	86(58,125)	Reference	Reference	Reference
- Primary	1131	179	161(138,188)	0.95(0.65,1.41)	0.96(0.63,1.46)	0.97(0.63,1.50)
- Secondary	1622	174	107(91,125)	0.85(0.59,1.22)	0.84(0.57,1.24)	0.87(0.59,1.28)
- College/ Uni	356	29	81(52,124)	0.94(0.57,1.55)	0.95(0.57,1.55)	1.01(0.62,1.64)
**Risk factors for NCD**						
Smoking						
- Never	2396	256	107(92,124)	Reference	Reference	Reference
- Former	252	33	131(93,180)	1.20(0.85,1.69)	1.20(0.85,1.71)	1.15(0.79,1.66)
- Current	775	120	156(132,184)	1.19(0.93,1.52)	1.17(0.91,1.50)	1.19(0.91,1.55)
Diabetes						
- No	3202	354	111(98,125)	Reference	Reference	Reference
- Yes	216	54	248(194,313)	2.16(1.69,2.77)	2.20(1.69,2.84)	2.34(1.81,3.03)
Hypertension						

For peer review only

- No	1670	179	108(92,126)	Reference	Reference	Reference
- Yes	1737	229	132(113,153)	1.08(0.90,1.29)	1.06(0.88,1.28)	1.11(0.92,1.34)
Alcohol						
- Never	1335	117	87(73,104)	Reference	Reference	Reference
- Former	1520	221	147(125,171)	1.18(0.95,1.48)	1.18(0.94,1.49)	1.15(0.90,1.46)
- Current	559	70	125(100,157)	1.15(0.86,1.53)	1.18(0.87,1.60)	1.08(0.77,1.51)
BMI						
- Underweight	468	99	216(169,271)	1.55(1.20,2.00)	1.57(1.22,2.02)	1.60(1.24,2.07)
- Normal	1697	199	117(101,136)	Reference	Reference	Reference
- Overweight	779	66	86(66,111)	0.90(0.69,1.18)	0.87(0.66,1.14)	0.81(0.62, 1.05)
- Obese	447	32	73(52,100)	0.89(0.63,1.25)	0.86(0.59,1.24)	0.83(0.57,1.20)

For peer review only

Table 5: Risk of mortality during 6 years of follow up by the presence of specific eye diseases at baseline

	No at risk	Deaths	Risk per 1,000/6yrs (95%CI)	Age-sex-adjusted Risk Ratio
Cataract present				
- No	1921	142	72.5(59.1,88.6)	Reference
- Yes	1478	265	181.3(161.6,202.8)	1.26(0.95,1.66)
Cataract and VI present (<6/18)				
- No	3103	313	100.7(87.5,115.5)	Reference
- Yes	296	94	322(267.3,381.8)	1.57(1.24,1.98)
AMD present				
- No	2270	225	99.2(83.3,117.8)	Reference
- Yes	319	57	183.4(141.3,234.5)	1.34(0.99,1.81)
AMD and VI present (<6/18)				
- No	2529	265	105.5(89.9,123.4)	Reference
- Yes	60	17	282.7(178.5,416.8)	1.42(0.91,2.22)
DR present				

For peer review only

- No	2513	264	105.7(90.4,123.3)	Reference
- Yes	55	18	318.4(199.4,467.0)	3.18(1.98,5.09)
DR and VI present (<6/18)				
- No	2563	280	109.9(94.1,128.0)	Reference
- Yes	5	2	401.6(37.5,920.4)	2.54(0.57,11.36)

For peer review only

References

1. Bourne RRA, Flaxman SR, Braithwaite T, et al. Magnitude, temporal trends, and projections of the global prevalence of blindness and distance and near vision impairment: a systematic review and meta-analysis. *Lancet Glob Health* 2017;5(9):e888-e97. doi: 10.1016/S2214-109X(17)30293-0 [published Online First: 2017/08/07]
2. Banks LM, Polack S. The Economic Costs of Exclusion and Gains of Inclusion of People with Disabilities. London: International Centre for Evidence in Disability, 2014.
3. Ribeiro MV, Hasten-Reiter Junior HN, Ribeiro EA, et al. Association between visual impairment and depression in the elderly: a systematic review. *Arq Bras Oftalmol* 2015;78(3):197-201. doi: 10.5935/0004-2749.20150051 [published Online First: 2015/07/30]
4. Polack S, Kuper H, Mathenge W, et al. Cataract visual impairment and quality of life in a Kenyan population. *The British journal of ophthalmology* 2007;91(7):927-32. doi: 10.1136/bjo.2006.110973 [published Online First: 2007/02/03]
5. Polack S, Kuper H, Wadud Z, et al. Quality of life and visual impairment from cataract in Satkhira district, Bangladesh. *The British journal of ophthalmology* 2008;92(8):1026-30. doi: 10.1136/bjo.2007.134791 [published Online First: 2008/07/26]
6. Clemons TE, Kurinij N, Sperduto RD. Associations of mortality with ocular disorders and an intervention of high-dose antioxidants and zinc in the Age-Related Eye Disease Study: AREDS Report No. 13. *Archives of ophthalmology (Chicago, Ill : 1960)* 2004;122(5):716-26. doi: 10.1001/archopht.122.5.716 [published Online First: 2004/05/12]
7. Freeman EE, Egleston BL, West SK, et al. Visual acuity change and mortality in older adults. *Investigative ophthalmology & visual science* 2005;46(11):4040-5. doi: 10.1167/iovs.05-0687 [published Online First: 2005/10/27]
8. Klein R, Klein BE, Moss SE. Age-related eye disease and survival. The Beaver Dam Eye Study. *Archives of ophthalmology (Chicago, Ill : 1960)* 1995;113(3):333-9. [published Online First: 1995/03/01]
9. Lam BL, Lee DJ, Gomez-Marin O, et al. Concurrent visual and hearing impairment and risk of mortality: the National Health Interview Survey. *Archives of ophthalmology (Chicago, Ill : 1960)* 2006;124(1):95-101. doi: 10.1001/archopht.124.1.95 [published Online First: 2006/01/13]
10. Thiagarajan M, Evans JR, Smeeth L, et al. Cause-specific visual impairment and mortality: results from a population-based study of older people in the United Kingdom. *Archives of ophthalmology (Chicago, Ill : 1960)* 2005;123(10):1397-403. doi: 10.1001/archopht.123.10.1397 [published Online First: 2005/10/13]
11. Karpa MJ, Mitchell P, Beath K, et al. Direct and indirect effects of visual impairment on mortality risk in older persons. *Archives of ophthalmology (Chicago, Ill : 1960)* 2009;127(10):1347-53. doi: 10.1001/archophthalmol.2009.240 [published Online First: 2009/10/14]

For peer review only

12. Wang JJ, Mitchell P, Simpson JM, et al. Visual impairment, age-related cataract, and mortality. *Archives of ophthalmology (Chicago, Ill : 1960)* 2001;119(8):1186-90. [published Online First: 2001/08/14]
13. Mitoku K, Masaki N, Ogata Y, et al. Vision and hearing impairments, cognitive impairment and mortality among long-term care recipients: a population-based cohort study. *BMC geriatrics* 2016;16:112. doi: 10.1186/s12877-016-0286-2 [published Online First: 2016/05/29]
14. Foong AW, Fong CW, Wong TY, et al. Visual acuity and mortality in a chinese population. The Tanjong Pagar Study. *Ophthalmology* 2008;115(5):802-7. doi: 10.1016/j.ophtha.2007.04.066 [published Online First: 2007/09/04]
15. Siantar RG, Cheng CY, Gemmy Cheung CM, et al. Impact of Visual Impairment and Eye diseases on Mortality: the Singapore Malay Eye Study (SiMES). *Scientific reports* 2015;5:16304. doi: 10.1038/srep16304 [published Online First: 2015/11/10]
16. Li Z, Sun D, Liu P, et al. Visual impairment and mortality in a rural adult population (the Southern Harbin eye study). *Ophthalmic epidemiology* 2011;18(2):54-60. doi: 10.3109/09286586.2010.545503 [published Online First: 2011/03/16]
17. Khanna RC, Murthy GV, Giridhar P, et al. Cataract, visual impairment and long-term mortality in a rural cohort in India: the Andhra Pradesh Eye Disease Study. *PloS one* 2013;8(10):e78002. doi: 10.1371/journal.pone.0078002 [published Online First: 2013/11/28]
18. Cong R, Zhou B, Sun Q, et al. Smoking and the risk of age-related macular degeneration: a meta-analysis. *Ann Epidemiol* 2008;18(8):647-56. doi: 10.1016/j.annepidem.2008.04.002
19. Organisation WH. World Report on Disability. Geneva: World Health Organisation 2011.
20. Pathai S, Shiels PG, Weiss HA, et al. Ocular parameters of biological ageing in HIV-infected individuals in South Africa: relationship with chronological age and systemic biomarkers of ageing. *Mech Ageing Dev* 2013;134(9):400-6. doi: 10.1016/j.mad.2013.08.002
21. Liljas AEM, Carvalho LA, Papachristou E, et al. Self-reported vision impairment and incident prefrailty and frailty in English community-dwelling older adults: findings from a 4-year follow-up study. *J Epidemiol Community Health* 2017;71(11):1053-58. doi: 10.1136/jech-2017-209207 [published Online First: 2017/08/12]
22. Cimarolli VR, Jopp DS. Sensory impairments and their associations with functional disability in a sample of the oldest-old. *Qual Life Res* 2014;23(7):1977-84. doi: 10.1007/s11136-014-0657-0 [published Online First: 2014/04/01]
23. Bastawrous A, Mathenge W, Peto T, et al. The Nakuru eye disease cohort study: methodology & rationale. *BMC Ophthalmol* 2014;14(1):60. doi: 10.1186/1471-2415-14-60
24. Turner AG, Magnani RJ, Shuaib M. A not quite as quick but much cleaner alternative to the Expanded Programme on Immunization (EPI) Cluster Survey design. *Int J Epidemiol* 1996;25(1):198-203. [published Online First: 1996/02/01]

For peer review only

25. Kuper H, Polack S, Eusebio C, et al. A case-control study to assess the relationship between poverty and visual impairment from cataract in Kenya, the Philippines, and Bangladesh. *PLoS Med* 2008;5(12):e244.
26. Seaman SR, White IR. Review of inverse probability weighting for dealing with missing data. *Statistical methods in medical research* 2013;22(3):278-95. doi: 10.1177/0962280210395740
27. Agrawal N, Kalaivani M, Gupta SK, et al. Association of blindness and hearing impairment with mortality in a cohort of elderly persons in a rural area. *Indian journal of community medicine : official publication of Indian Association of Preventive & Social Medicine* 2011;36(3):208-12. doi: 10.4103/0970-0218.86522 [published Online First: 2011/11/18]
28. Fisher D, Li CM, Chiu MS, et al. Impairments in hearing and vision impact on mortality in older people: the AGES-Reykjavik Study. *Age and ageing* 2014;43(1):69-76. doi: 10.1093/ageing/aft122 [published Online First: 2013/09/03]
29. Kuang TM, Tsai SY, Liu CJ, et al. The association of visual impairment and 3-year mortality among the elderly in Taiwan: the Shihpai Eye Study. *Journal of the Chinese Medical Association : JCMA* 2015;78(3):177-81. doi: 10.1016/j.jcma.2014.11.006 [published Online First: 2015/01/27]
30. Mathenge W, Foster A, Kuper H. Urbanization, ethnicity and cardiovascular risk in a population in transition in Nakuru, Kenya: a population-based survey. *BMC Public Health* 2010;10:569. doi: 10.1186/1471-2458-10-569 [published Online First: 2010/09/24]
31. Knudtson MD, Klein BE, Klein R. Age-related eye disease, visual impairment, and survival: the Beaver Dam Eye Study. *Archives of ophthalmology (Chicago, Ill : 1960)* 2006;124(2):243-9. doi: 10.1001/archopht.124.2.243 [published Online First: 2006/02/16]
32. Zhu XR, Zhang YP, Bai L, et al. Prediction of risk of diabetic retinopathy for all-cause mortality, stroke and heart failure: Evidence from epidemiological observational studies. *Medicine (Baltimore)* 2017;96(3):e5894. doi: 10.1097/MD.0000000000005894
33. Cugati S, Cumming RG, Smith W, et al. Visual impairment, age-related macular degeneration, cataract, and long-term mortality: the Blue Mountains Eye Study. *Archives of ophthalmology (Chicago, Ill : 1960)* 2007;125(7):917-24. doi: 10.1001/archopht.125.7.917 [published Online First: 2007/07/11]
34. Song E, Sun H, Xu Y, et al. Age-related cataract, cataract surgery and subsequent mortality: a systematic review and meta-analysis. *PloS one* 2014;9(11):e112054. doi: 10.1371/journal.pone.0112054
35. Borger PH, van Leeuwen R, Hulsman CA, et al. Is there a direct association between age-related eye diseases and mortality? The Rotterdam Study. *Ophthalmology* 2003;110(7):1292-6. doi: 10.1016/s0161-6420(03)00450-0 [published Online First: 2003/07/18]
36. Nucci C, Cedrone C, Culasso F, et al. Association between lens opacities and mortality in the Priverno Eye Study. *Graefe's archive for clinical and experimental ophthalmology = Albrecht von Graefes Archiv fur klinische und experimentelle Ophthalmologie* 2004;242(4):289-94. doi: 10.1007/s00417-003-0837-z [published Online First: 2004/01/24]

For peer review only - http://bmjopen.bmj.com/site/about/guidelines.xhtml

37. Minassian DC, Mehra V, Johnson GJ. Mortality and cataract: findings from a population-based longitudinal study. *Bulletin of the World Health Organization* 1992;70(2):219-23. [published Online First: 1992/01/01]
38. Hennis A, Wu SY, Li X, et al. Lens opacities and mortality : the Barbados Eye Studies. *Ophthalmology* 2001;108(3):498-504. [published Online First: 2001/03/10]
39. Williams SL, Ferrigno L, Mora P, et al. Baseline cataract type and 10-year mortality in the Italian-American Case-Control Study of age-related cataract. *American journal of epidemiology* 2002;156(2):127-31. [published Online First: 2002/07/16]
40. Wong TY, Tikellis G, Sun C, et al. Age-related macular degeneration and risk of coronary heart disease: the Atherosclerosis Risk in Communities Study. *Ophthalmology* 2007;114(1):86-91. doi: 10.1016/j.ophtha.2006.06.039 [published Online First: 2007/01/03]
41. Fisher DE, Jonasson F, Eiriksdottir G, et al. Age-related macular degeneration and mortality in community-dwelling elders: the age, gene/environment susceptibility Reykjavik study. *Ophthalmology* 2015;122(2):382-90. doi: 10.1016/j.ophtha.2014.08.006 [published Online First: 2014/09/30]
42. Gopinath B, Liew G, Burlutsky G, et al. Age-related macular degeneration and risk of total and cause-specific mortality over 15 years. *Maturitas* 2016;84:63-7. doi: 10.1016/j.maturitas.2015.11.001 [published Online First: 2015/11/26]
43. Pedula KL, Coleman AL, Yu F, et al. Age-related macular degeneration and mortality in older women: the study of osteoporotic fractures. *Journal of the American Geriatrics Society* 2015;63(5):910-7. doi: 10.1111/jgs.13405 [published Online First: 2015/05/06]
44. Akbari M, Akbari S, Pasquale LR. The association of primary open-angle glaucoma with mortality: a meta-analysis of observational studies. *Archives of ophthalmology (Chicago, Ill : 1960)* 2009;127(2):204-10. doi: 10.1001/archophthalmol.2008.571
45. Boerma T. Moving towards better cause of death registration in Africa and Asia. *Glob Health Action* 2014;7:25931. doi: 10.3402/gha.v7.25931 [published Online First: 2014/11/08]
46. Reidy A, Minassian DC, Desai P, et al. Increased mortality in women with cataract: a population based follow up of the North London Eye Study. *The British journal of ophthalmology* 2002;86(4):424-8. [published Online First: 2002/03/27]
47. Lee DJ, Gomez-Marin O, Lam BL, et al. Glaucoma and survival: the National Health Interview Survey 1986-1994. *Ophthalmology* 2003;110(8):1476-83. doi: 10.1016/s0161-6420(03)00408-1 [published Online First: 2003/08/15]
48. Lee AJ, Wang JJ, Kifley A, et al. Open-angle glaucoma and cardiovascular mortality: the Blue Mountains Eye Study. *Ophthalmology* 2006;113(7):1069-76. doi: 10.1016/j.ophtha.2006.02.062 [published Online First: 2006/07/04]
49. Mathenge W, Bastawrous A, Foster A, et al. The Nakuru posterior segment eye disease study: methods and prevalence of blindness and visual impairment in Nakuru, Kenya. *Ophthalmology* 2012;119(10):2033-9. doi: 10.1016/j.ophtha.2012.04.019 [published Online First: 2012/06/23]

For peer review only

Web Table 1. 6-Year adjusted mortality risk by level of VI among the Nakuru Eye Disease Cohort Study Participants, stratified by age and gender (Unweighted)

	Overall		Male		Female		<60 years		≥60 years	
	N	Risk per 1,000/6yrs (95%CI)	N	Risk per 1,000/6yrs (95%CI)	N	Risk per 1,000/6yrs (95%CI)	N	Risk per 1,000/6yrs (95%CI)	N	Risk per 1,000/6yrs (95%CI)
Visual acuity at baseline (better eye presenting)										
All individuals	409 / 3441	119(106,134)	236 / 1656	143(124,164)	173 / 1785	97(83,113)	86 / 1503	57(46,71)	323 / 1938	167(150,185)
Normal (≥6/12)	280 / 2901	97(84,111)	162 / 1378	118(98,140)	118 / 1523	78(64,93)	78 / 1420	55(44,69)	202 / 1481	136(118,157)
Near Normal (<6/12-≥6/18)	27 / 170	159(109,226)	14 / 84	167(104,256)	13 / 86	151(87,250)	3 / 27	111(34,309)	24 / 143	168(115,238)
VI (<6/18-≥6/60)	77 / 275	280(234,331)	45 / 142	317(252,390)	32 / 133	241(172,325)	3 / 31	97(30,268)	74 / 244	303(250,363)
SVI (<6/60-≥3/60)	4 / 16	250(83,552)	2 / 10	200(37,622)	2 / 6	333(42,851)	2 / 2	-	2 / 14	143(28,490)
Blind (<3/60)	19 / 50	380(242,541)	13 / 30	433(246,642)	6 / 20	300(129,553)	0 / 6	-	19 / 44	433(275,604)
Any VI (<6/18)	100 / 341	293(245,347)	60 / 182	330(268,398)	40 / 159	252(184,333)	5 / 39	128(52,281)	95 / 302	315(260,375)

For peer review only

STROBE 2007 (v4) Statement—Checklist of items that should be included in reports of *cohort studies*

Section/Topic	Item #	Recommendation	Reported on page #
**Title and abstract**	1	(*a*) Indicate the study's design with a commonly used term in the title or the abstract	1, 2
		(*b*) Provide in the abstract an informative and balanced summary of what was done and what was found	2
**Introduction**			
Background/rationale	2	Explain the scientific background and rationale for the investigation being reported	5
Objectives	3	State specific objectives, including any prespecified hypotheses	5
**Methods**			
Study design	4	Present key elements of study design early in the paper	6
Setting	5	Describe the setting, locations, and relevant dates, including periods of recruitment, exposure, follow-up, and data collection	6
Participants	6	(*a*) Give the eligibility criteria, and the sources and methods of selection of participants. Describe methods of follow-up	6, 7
		(*b*) For matched studies, give matching criteria and number of exposed and unexposed	-
Variables	7	Clearly define all outcomes, exposures, predictors, potential confounders, and effect modifiers. Give diagnostic criteria, if applicable	6, 7
Data sources/ measurement	8*	For each variable of interest, give sources of data and details of methods of assessment (measurement). Describe comparability of assessment methods if there is more than one group	7
Bias	9	Describe any efforts to address potential sources of bias	8
Study size	10	Explain how the study size was arrived at	6
Quantitative variables	11	Explain how quantitative variables were handled in the analyses. If applicable, describe which groupings were chosen and why	7,8
Statistical methods	12	(*a*) Describe all statistical methods, including those used to control for confounding	7,8
		(*b*) Describe any methods used to examine subgroups and interactions	7,8
		(*c*) Explain how missing data were addressed	7,8
		(*d*) If applicable, explain how loss to follow-up was addressed	7,8
		(*e*) Describe any sensitivity analyses	7,8
**Results**			

For peer review only

Participants	13*	(a) Report numbers of individuals at each stage of study—eg numbers potentially eligible, examined for eligibility, confirmed eligible, included in the study, completing follow-up, and analysed	10
		(b) Give reasons for non-participation at each stage	10
		(c) Consider use of a flow diagram	-
Descriptive data	14*	(a) Give characteristics of study participants (eg demographic, clinical, social) and information on exposures and potential confounders	10
		(b) Indicate number of participants with missing data for each variable of interest	In tables
		(c) Summarise follow-up time (eg, average and total amount)	10
Outcome data	15*	Report numbers of outcome events or summary measures over time	10
Main results	16	(*a*) Give unadjusted estimates and, if applicable, confounder-adjusted estimates and their precision (eg, 95% confidence interval). Make clear which confounders were adjusted for and why they were included	Tables 1 and 2
		(*b*) Report category boundaries when continuous variables were categorized	All tables
		(*c*) If relevant, consider translating estimates of relative risk into absolute risk for a meaningful time period	-
Other analyses	17	Report other analyses done—eg analyses of subgroups and interactions, and sensitivity analyses	10
**Discussion**			
Key results	18	Summarise key results with reference to study objectives	12
**Limitations**			
Interpretation	20	Give a cautious overall interpretation of results considering objectives, limitations, multiplicity of analyses, results from similar studies, and other relevant evidence	13
Generalisability	21	Discuss the generalisability (external validity) of the study results	12
**Other information**			
Funding	22	Give the source of funding and the role of the funders for the present study and, if applicable, for the original study on which the present article is based	3

*Give information separately for cases and controls in case-control studies and, if applicable, for exposed and unexposed groups in cohort and cross-sectional studies.

Note: An Explanation and Elaboration article discusses each checklist item and gives methodological background and published examples of transparent reporting. The STROBE checklist is best used in conjunction with this article (freely available on the Web sites of PLoS Medicine at http://www.plosmedicine.org/, Annals of Internal Medicine at http://www.annals.org/, and Epidemiology at http://www.epidem.com/). Information on the STROBE Initiative is available at www.strobe-statement.org.

For peer review only
